# Acoustic and prosodic speech features reflect physiological stress but not isolated negative affect: a multi-paradigm study on psychosocial stressors

**DOI:** 10.1038/s41598-024-55550-3

**Published:** 2024-03-06

**Authors:** Mitchel Kappen, Gert Vanhollebeke, Jonas Van Der Donckt, Sofie Van Hoecke, Marie-Anne Vanderhasselt

**Affiliations:** 1 Department of Head and Skin, Department of Psychiatry and Medical Psychology, Ghent University, University Hospital Ghent (UZ Ghent), Ghent, Belgium; 2 Ghent Experimental Psychiatry (GHEP) Lab, Ghent University, Ghent, Belgium; 3 Department of Experimental Clinical and Health Psychology, Ghent University, Ghent, Belgium; 4 IDLab, Ghent University - Imec, Ghent, Belgium; 5 Department of Electronics and Information Systems, Ghent University, Ghent, Belgium

**Keywords:** Psychology, Predictive markers

## Abstract

Heterogeneity in speech under stress has been a recurring issue in stress research, potentially due to varied stress induction paradigms. This study investigated speech features in semi-guided speech following two distinct psychosocial stress paradigms (Cyberball and MIST) and their respective control conditions. Only negative affect increased during Cyberball, while self-reported stress, skin conductance response rate, and negative affect increased during MIST. Fundamental frequency (F0), speech rate, and jitter significantly changed during MIST, but not Cyberball; HNR and shimmer showed no expected changes. The results indicate that observed speech features are robust in semi-guided speech and sensitive to stressors eliciting additional physiological stress responses, not solely decreases in negative affect. These differences between stressors may explain literature heterogeneity. Our findings support the potential of speech as a stress level biomarker, especially when stress elicits physiological reactions, similar to other biomarkers. This highlights its promise as a tool for measuring stress in everyday settings, considering its affordability, non-intrusiveness, and ease of collection. Future research should test these results' robustness and specificity in naturalistic settings, such as freely spoken speech and noisy environments while exploring and validating a broader range of informative speech features in the context of stress.

## Introduction

Stress is a physiological and psychological response to internal or external stimuli that are perceived as threatening to an individual’s well-being^1,2^. Whether it be personal or professional, acute or chronic, stress is a common aspect of modern life that impacts people of all ages and backgrounds. Acute stress is a normal part of the human experience and can be adaptive in the short term by enabling individuals to respond to challenges and adapt to their environment^2^. However, when stress becomes chronic, it can have serious and long-lasting effects on an individual’s physical and mental health, such as cardiovascular disease, cognitive impairment, depression, anxiety, and other (mental) health disorders^3,4^. As a result, accurately measuring and regularly monitoring stress levels is crucial for maintaining optimal health and well-being^5,6^.

Given chronic stress's effects on mental and physical health, various methods have been developed for assessing peoples’ stress levels including physiological, self-report, and behavioral methods^7^. While each method has advantages, they also have unresolved limitations, such as cost, validity, intrusiveness, or lack of accuracy in natural settings^8^. Consequently, speech has been suggested as a non-intrusive and cost-effective method capable of measuring stress over extended periods. Speech recordings can be obtained from various sources, such as phone calls or meetings, without the need for specialized equipment, making it cost-effective and allowing for data collection in naturalistic settings, which reduces intrusiveness^8–10^.

Acoustic changes in speech have been observed in response to (acute) stress^9,11,12^. However, whereas most former analyses were done in studies that used either voice actors or read-out-loud speech paradigms, it is important to shift towards evaluating the potential of freely spoken speech as it would occur in daily life^13^ to not limit the ecological validity of the results. In addition, whereas former studies rarely used validated stress paradigms or failed to validate the stress experience of participants, our recent studies addressed these limitations by utilizing validated stress induction techniques and gathering self-reports. However, they are still limited by (1) the use of one single stress induction paradigm and (2) read-out-loud speech^10,11^.

In the current study, we focus on a key set of features that, although varying in the frequency of appearance in the literature, have consistently demonstrated their relevance across studies. We refer to these features as acoustic (physical properties of speech) and prosodic (suprasegmental aspects of speech contributing to the overall rhythm, intonation, and stress patterns), with all chosen features belonging to either one or both categories. These include the Fundamental Frequency (F0), a measure of the vocal cord’s vibration frequency, that generally increases with stress^9,11,12^, jitter (vocal frequency variation) and shimmer (vocal intensity variation) which have been observed to decrease due to stress^9,11,12^, and the Harmonics-to-Noise Ratio (HNR; relative amount of noise in comparison to harmonics in the voice) which has been shown to decrease in the context of a physical stressor (any physical event or stimulus that elicits stress) and has mixed results in the context of psychological stress^9,11,14–16^. Additionally, we will investigate the effect of stress on changes in speech rate (talking speed) which has been shown to increase during stress in free speech samples^9,17,18^.

While previous studies have identified links between specific (acoustic) features of speech and stress, more research is needed to fully understand the current heterogeneity, robustness, and sensitivity of these relationships^9,10,12^. This can only be done if we do not limit our studies to single stress paradigms, especially considering that different stressors used in these paradigms elicit different stress responses. Therefore, exposing participants to different stress paradigms, but with similar experimental setups (i.e., active control task vs stress task), will allow us to better understand the basis of the observed effects on speech under stress. We aim to understand whether the observed changes in speech features that occur are related to one’s changes in mood (e.g., increased negative affect) or to physiological reactions (by activation of the hypothalamic-pituitary-adrenocortical (HPA) axis), by using two well-established stress induction paradigms that specifically elicit these changes. We employed the Cyberball^19^ and the Montreal Imaging Stress Task (MIST)^20^ paradigms to address these limitations and further uncover the sensitivity and robustness of speech features under stress. Both paradigms use a psychosocial stressor, include an active control condition, and unscripted speech will be collected by having participants describe screenshots from the paradigm. See Van Der Donckt and colleagues for a thorough comparison of speech styles and considerations in speech collection paradigms^13^.

The main difference between these two paradigms is that the Cyberball induces stress in the form of feelings of negative mood by means of *ostracism* due to excluding the participant from the task^19,21^, whereas the MIST induces stress by adding components of *social evaluative threat* (SET) to a cognitively challenging task^20^. Ostracism has been shown to worsen one’s mood but is mostly limited to psychological responses and does not show a neuroendocrine (cortisol) response^21–23^, whereas SET elicits a strong physiological, neuroendocrine (cortisol) response in addition to a decreased mood^7,24–26^.

### Research objectives and hypotheses

We will gauge the stress response based on increased skin conductance response rate (SCRR), as well as self-reports on increased experienced stress and negative affect during the stress block as compared to the control block. Moreover, this is the first study to use a picture-describe paradigm to capture semi-guided speech that closely resembles natural speech in order to yield ecologically valid results. For more details, see Van Der Donckt and colleagues^13^. *Negative Affect*. We expect increases in Negative Affect after the stress blocks compared to the control blocks for both paradigms. *Self-reported Stress*. We expect increased self-reported stress during the stress block for the MIST. However, we do not expect increases in self-reported stress for the Cyberball, as its effect is inconsistent and strongly mediated by traits such as the need to belong, limiting the observance of this effect in a general population^27^. *Skin Conductance Response Rate (SCRR).* We expect an increase in SCRR during the stress block for the MIST, but not for the Cyberball. *Speech features.* We expect similar results for speech features as observed in earlier studies that used read-out-loud protocols^9,11,12^ for the MIST. That is, increases in Fundamental Frequency (F0)^9,11,12^ and measures of changes in speech rate^9,18^, decreases in Jitter^9,12^ and Shimmer^9,11,12^, and changes in Harmonics to Noise (HNR; added noise in the voice), but the direction is unclear due to mixed results in the context of psychological stressors^9,11,14–16^. In the Cyberball paradigm, the occurrence and direction of significant speech feature changes will reveal the sensitivity and heterogeneity of speech as a biomarker for (psychological) stress. Considering the expected difference in the stress reaction in the Cyberball (negative mood) compared to the MIST (negative mood + physiological reaction), the occurrence of significant changes in speech features would show that speech is responsive to mere changes in mood due to stress (therefore occurring in both paradigms). A lack of changes in speech features would, however, illustrate that speech (features) are merely related to physiological stress responses and therefore follow the patterns (i.e., effects in MIST, but not in Cyberball) observed in other biomarkers such as cortisol^7,25,26^. Lastly, it is possible that different speech features have varying sensitivity, where some might just be responsive to combined mood and physiological stress responses, and others might be responsive to mere changes in mood. For example, one of the most homogeneously reported speech features to change under stress is F0, which could indicate that this is a sensitive feature to any change in experienced stress (i.e., mood or physiological) since it occurs in many different studies and stress paradigms. Other features have shown to be more heterogeneous (e.g., HNR, Jitter), which could be explained by the use of different paradigms and stressors. The combination of two different stress induction paradigms, which elicit different stress responses by calling on different psychological constructs (Cyberball; ostracism, MIST; social evaluative threat), tested in the same group of participants, will give unique insights into the robustness (by using semi-guided speech), sensitivity (by comparing a mood only to a mood plus physiological stress reaction), and up to this point heterogeneity (by comparing two commonly used stress paradigms) in a variety of speech features under stress.

## Results

**Figure 1:**
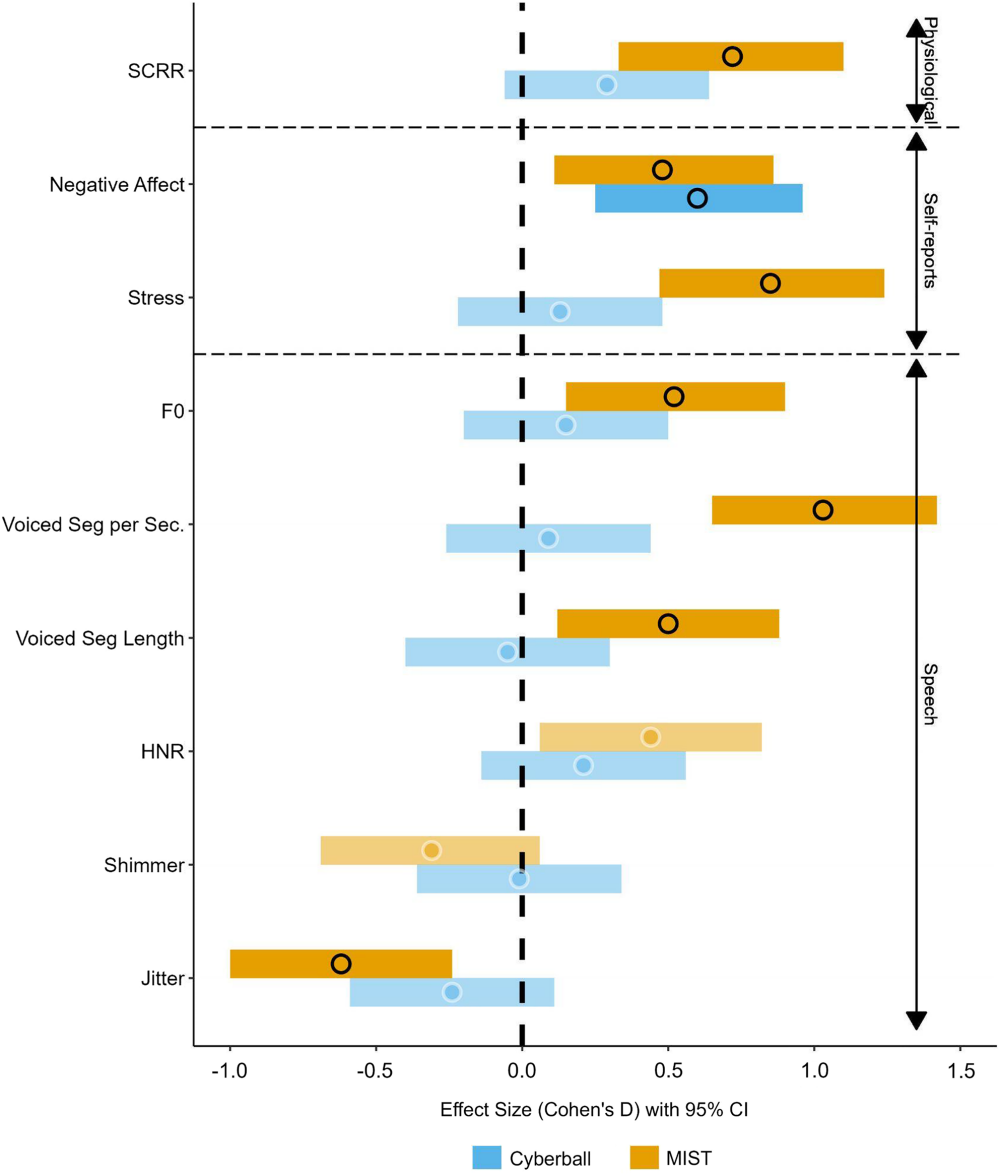
Forest plot of effect sizes and confidence intervals for all control-stress comparisons per paradigm. *Note* Effect sizes (dots; Cohen’s D) and 95% CIs (bars) for each control vs stress task comparison per stress induction paradigm (Cyberball—MIST). Dependent variables are grouped on their categories (i.e., physiological, self-report, speech) and are FDR corrected within their respective categories. Dots are circled black and ranges are saturated (i.e. non-transparent) if a significant effect is observed after correction.

Throughout the different paradigms, we focus in our analyses on three main modalities of which two are already more validated in literature (i.e., self-reports and physiological measures) and one is our novel addition to the state-of-the-art (i.e., speech). The models reported will contain the dependent variable, taskPhase (control vs stress task), taskType (MIST vs Cyberball), Gender (man vs woman) if showing to be a significant contributor, and ‘(1|ID)’; a random intercept for each participant. Per category (i.e., self-reports, physiological, and speech), for each feature, we will only describe the pairwise comparisons of stress vs control block for each individual paradigm as these are directly related to our research questions. All effect sizes and corresponding 95% confidence intervals for the control-stress comparisons per feature, per paradigm, are also displayed in Fig. 1. Full model information and corresponding statistics are described in the analyses section of the supplemental materials.

### Physiological

#### Skin conductance response rate (SCRR)

A significant increase in *SCRR* was observed during the stress task in the MIST, *b* = 1.30, *SE* = 0.34, *t* = 3.77, *p* < 0.001, *d* = 0.72., 95% CI [0.33, 1.10], but not in the Cyberball, *b* = 0.53, *SE* = 0.32, *t* = 1.66, *p* = 0.098, *d* = 0.29, 95% CI [− 0.06, 0.64].

### Self-reports

#### Negative affect

A significant increase in *Negative Affect* was observed during the stress task in the MIST, *b* = 4.29, *SE* = 1.69, *t* = 2.54, *p* = 0.016, *d* = 0.48., 95% CI [0.11, 0.86], as well as in the Cyberball, *b* = 5.37, *SE* = 1.57, *t* = 3.42, *p* = 0.002, *d* = 0.60, 95% CI [0.25, 0.96].

#### Stress

A significant increase in self-reported *Stress* was observed during the stress task in the MIST, *b* = 17.26, *SE* = 3.84, *t* = 4.49, *p* < 0.001, *d* = 0.85., 95% CI [0.47, 1.24], but not in the Cyberball, *b* = 2.60, *SE* = 3.58, *t* = 0.73, *p* = 0.469, *d* = 0.13, 95% CI [− 0.22, 0.48].

### Speech/acoustic

#### Fundamental frequency (F0)

A significant increase in *F0* was observed during the stress task in the MIST, *b* = 0.42, *SE* = 0.15, *t* = 2.76, *p* = 0.026, *d* = 0.52., 95% CI [0.15, 0.90], but not in the Cyberball, *b* = 0.12, *SE* = 0.14, *t* = 0.85, *p* = 0.531, *d* = 0.15, 95% CI [− 0.20, 0.50].

#### Voiced segments per second

A significant increase in *voiced segments per second* was observed during the stress task in the MIST, *b* = 0.23, *SE* = 0.04, *t* = 5.44, *p* < 0.001, *d* = 1.03., 95% CI [0.65, 1.42], but not in the Cyberball, *b* = 0.02, *SE* = 0.04, *t* = 0.52, *p* = 0.729, *d* = 0.09, 95% CI [− 0.26, 0.44].

#### Voiced segment length

A significant increase in *voiced segment length* was observed during the stress task in the MIST, *b* = 0.01, *SE* = 0.005, *t* = 2.62, *p* = 0.029, *d* = 0.50., 95% CI [0.12, 0.88], but not in the Cyberball, *b* = − 0.001, *SE* = 0.005, *t* = − 0.27, *p* = 0.858, *d* = − 0.05, 95% CI [− 0.40, 0.30].

#### Harmonics-to-noise ratio (HNR)

No significant change in *HNR* was observed during the stress task in the MIST, *b* = 0.21, *SE* = 0.09, *t* = 2.31, *p* = 0.053, *d* = 0.44., 95% CI [0.06, 0.82], nor in the Cyberball, *b* = 0.10, *SE* = 0.08, *t* = 1.19, *p* = 0.354, *d* = 0.21, 95% CI [− 0.14, 0.56].

#### Shimmer

No change in *Shimmer* was observed during the stress task in the MIST, *b* = − 0.02, *SE* = 0.01, *t* = − 1.65, *p* = 0.201, *d* = − 0.31., 95% CI [− 0.69, 0.06], nor in the Cyberball, *b* = 0, *SE* = 0.01, *t* = − 0.07, *p* = 0.942, *d* = − 0.01, 95% CI [− 0.36, 0.34].

#### Jitter

A significant decrease in *Jitter* was observed during the stress task in the MIST, *b* = − 0.003, *SE* = 0.0009, *t* = − 3.28, *p* = 0.007, *d* = − 0.62., 95% CI [− 1.00, − 0.24], but not in the Cyberball, *b* = − 0.001, *SE* = 0.0009, *t* = − 1.38, *p* = 0.292, *d* = − 0.24, 95% CI [− 0.59, 0.11].

#### Correlations

In our endeavor to present a comprehensive overview of the interrelations among the speech features and stress measures utilized in this study, we conducted additional correlational analyses. These analyses serve to contextualize the complex relationships without drawing direct interpretative conclusions, thereby maintaining scientific completeness. We identified several significant correlations, for instance, direct correlations between self-reported stress and F0, Jitter, and HNR. Moreover, multiple significant correlations are observed between the different speech features and psychophysiological measures. The complete correlation table, which includes all speech features as well as self-reported stress (VAS_Stress) and psychophysiological measures (SCRR and HRV-RMSSD), can be found in Sect. 7.10 of the supplemental materials. We direct readers to this section for a detailed view of the correlation coefficients, which are visually represented to indicate both the strength (through color intensity) and direction (blue for positive, red for negative) of each significant relationship.

## Discussion

In this study, we aimed to gain insights into the robustness (by using semi-guided instead of read-out-loud speech), sensitivity (by comparing a mood only to a mood plus physiological stress reaction), and up to this point observed heterogeneity (by comparing two different commonly used stress paradigms) of the effects of stress on acoustic and prosodic speech features. On two different days, participants were exposed to two different stress induction paradigms (i.e., Cyberball and Montreal Imaging Stress Task; MIST) with an expected different stress reaction (i.e., Cyberball; changes in mood, MIST; changes in mood and physiological response). Both paradigms included an active control condition in order to isolate the effects of added stress on their speech. Speech samples were collected directly after each paradigm phase (i.e., control or stress phase) using a picture-describe paradigm (prompting participants to describe a screenshot from the paradigm) to capture semi-guided speech that closely resembles natural speech in order to yield ecologically valid results. For more details, see Van Der Donckt and colleagues^13^.

First, we used validated measures to gauge how the stress responses elicited by the different stress paradigms (Cyberball and MIST), differed. As such, we observed that when considering physiological responses, there was only an increase in skin conductance response rate (SCRR) during the stress phase of the MIST, but not for the Cyberball, which corresponds to our prior hypotheses. In addition to the physiological responses, we also assessed participants' moods using self-reported measures. In line with our expectations, we observed an increase in self-reported negative affect during the stress task of both paradigms. Moreover, participants only reported increased self-reported stress during the MIST. These results are in line with the literature, as previously mentioned. The Cyberball task affects one's mood due to feelings of ostracization, but it only elicits psychological responses and does not elicit a physiological, neuroendocrine stress response^21–23^. However, the MIST, using social evaluative threat by means of (negative) social comparison, elicits both physiological and neuroendocrine stress responses, alongside a decrease in mood^7,20,24–26^.

Several key acoustic features, described in the literature to be responsive to stress, were extracted from the speech samples (F0; fundamental frequency, HNR; harmonics-to-noise ratio, jitter, shimmer, speech rate, and voiced segment length). Prior results have been heterogeneous, which is possibly due to the use of many different paradigms and stressors which introduce noise rather than robustness in this new modality’s early, exploratory stages. We tackle this by doing explicit, side by side analysis of two often used stress paradigms in the same sample. In the current study, during the Cyberball task, none of the tested acoustic speech features changed significantly during the stress, compared to the control phase. On the other hand, however, all features except HNR and shimmer changed in the expected direction during the stress phase of the MIST. We observe increases in F0 (in line with previous literature^9,11,12^), speech rate and voiced segment length (in line with previous literature^9,17,18^), and a decrease in Jitter (in line with previous literature^9,12^).

The observed increase in HNR, related to stress in the MIST, did not survive multiple comparison corrections, which indicates that the observed effect was rather small. This is in line with the literature, as previous studies have reported mixed results or conflicting findings in HNR changes^9,14–16^. However, the observed effect in the current study follows the same direction as our former study, which did show a significant increase^11^. This consistency might be indicative of the true direction of the effect, despite the small effect size in the present study. Additionally, no decrease was observed for shimmer during the stress task, whereas mixed results have been observed in the literature^9,11,12^. The absence of a significant change in shimmer during the stress phase of the MIST can be related to two things. First, it could be related to the speech collection paradigm used in the current study. We used a semi-guided speech paradigm in which participants were shown a screenshot from the task they just completed and were prompted to describe it. For the MIST, that means participants were shown a mathematical puzzle, similar to the earlier task, which many participants would try and solve out loud. This speech follows a less natural flow than naturally spoken speech, and as such could affect the amount of changes in shimmer. Second, which is arguably related to the first, the absolute observed values for shimmer were rather low as compared to former studies^11^ indicating potential floor effects. However, it should be noted that the observed absolute shimmer values are consistently lower for the recordings in the Cyberball paradigm (see supplemental materials). Nonetheless, due to the presented images being different between the two speech collection paradigms, no formal comparisons between the two should be done, and our interpretations are limited to within-paradigm changes. The methodological choice, to have participants describe screenshots from the task rather than other, off-topic images, should also be noted as the study’s biggest limitation. The study’s objective was, first and foremost, to elicit stress using two different psychosocial stress induction paradigms. By having participants describe unrelated images directly after completing the task, it can be argued that they could be distracted from the stressor and thus decrease its potency, confounding the final results. As such, follow-up studies should collect speech samples using a standardized semi-guided speech paradigm consisting of validated images that are congruent to psychosocial paradigms, to describe and keep consistent throughout longitudinal designs as described in Van der Donckt and colleagues^13^. It should also be noted that the sample consisted of predominantly young adults, therefore possibly limiting the generalization of our results to the general population. Moreover, in addressing the effects of gender in our analyses, we have carefully considered gender as a fixed effect in our models where it was statistically justified to interpret our effects regardless of gender differences. We recognize that gender is a complex and multifaceted construct that intersects with a myriad of biological and social factors influencing stress responses. Our publicly available dataset allows for a more detailed investigation into these effects, which lay outside the scope of the current manuscript. We encourage researchers specifically interested in gender differences in stress responses to explore this dataset further.

The current study used two different psychosocial stress paradigms that are different in their stress responses (i.e., Cyberball; negative mood, MIST; negative mood + physiological reaction). As such, we were able to relate promising acoustic and prosodic speech features to these distinct stress responses. We demonstrate that most features that are described in the literature in relation to stress only changed in the MIST (social evaluative threat paradigm), and not in the Cyberball (ostracism paradigm). These results follow our observed changes in self-report and physiological measures and as such, we conclude that speech as a biomarker is indeed a promising method for detecting changes in stress levels. Speech is comparable to other validated methods (i.e., skin conductance response rate & self-reported stress) as illustrated by the observed effect sizes, in that it does not respond to mere changes in negative affect, but only when physiological changes occur.

This study is the first to demonstrate how speech features change due to different stress paradigms and corresponding stressors, using a within-participant design. These results might explain the current heterogeneity in the literature with regard to these speech features. We conclude that semi-freely spoken speech (features) are promising for stress detection, and are not affected by stressors that only evoke changes in negative mood. This further outlines its potential in real-world applications, where it appears increasingly promising in passive, remote, non-intrusive tracking of major stressors in daily life that can have severe health implications^28^.

## Conclusions

To conclude, we collected repeated semi-guided speech fragments from participants in two different psychosocial stress paradigms, both including active control conditions. We observed distinct stress reactions in the two paradigms through self-reports and psychophysiological responses. A change in self-reported negative affect during the Cyberball, and an additional physiological and self-reported stress reaction during the MIST were found. Similar effects (i.e., effect during MIST, but not during Cyberball) were found for most speech features of interest; F0, voiced segments per second, mean voiced segment length, and jitter, but not for HNR and shimmer. Therefore, we conclude that these effects are robust in (semi-)freely spoken speech (as compared to earlier studies using read-out-loud speech), and are sensitive to stressors that activate the HPA axis, but not to changes in negative affect alone. The difference in observed effects between the two stressors possibly explains the current heterogeneity in the literature. These results further solidify the potential use of speech as a biomarker for stress level assessment in everyday settings, given its affordability, non-intrusiveness, and ease of collection. Future studies should focus on further testing the robustness of these results in increasingly naturalistic settings, such as completely freely spoken speech and noisy environments while exploring a broader range of speech features that can be informative in the context of stress.

## Methods

### Participants

A convenience sample of 66 healthy subjects (13 women, 53 men, age M = 21.29, SD = 2.82) was recruited through social media. Upon registration, participants were checked for exclusion criteria (see supplemental material). The study was conducted in accordance with the declaration of Helsinki and received ethical approval from the Ghent University hospital ethical committee (registration number: B6702020000676). Another part of the study investigates the effects of (psychosocial) stressors on neural correlates. Results of electrophysiological correlates will be published elsewhere. Other collected data that were not part of the current paper’s research objectives will only be described in the supplemental materials.

All participants gave written informed consent before participating and were debriefed afterward on the true purpose of the study. A 40 Euro compensation fee was awarded upon completion of both testing days through bank transfer.

### Procedure

#### On-site experimental session

Participants completed online informed consent and trait questionnaires (beyond this paper’s scope) prior to two in-person experimental sessions, which were conducted in a dedicated room in the Department of Adult Psychiatry at Ghent University Hospital. At the start of the first on-site session, participants signed a paper consent form, and experimenters reviewed the cover story (see Cover Story).

**Figure 2:**
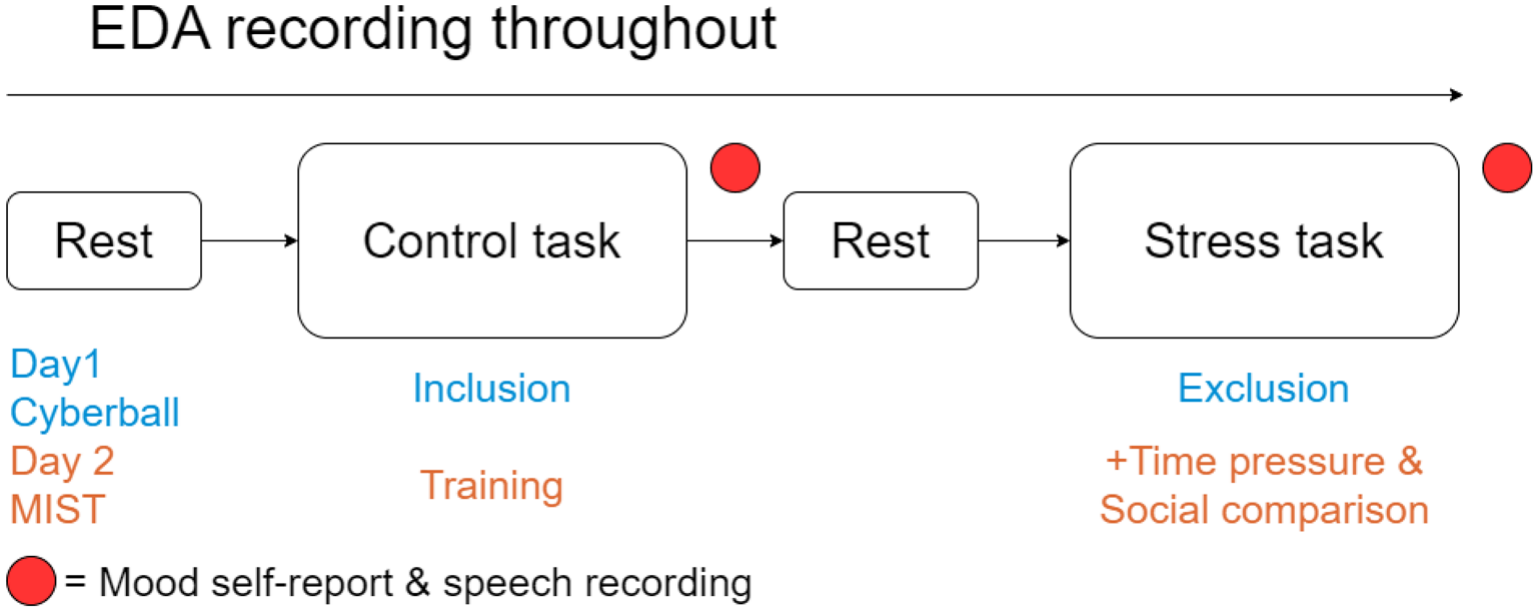
Flowchart of experimental design. *Note* The two days follow a similar structure except for the presented paradigm and respective control and stress tasks. EDA (electrodermal activity) is collected throughout the paradigm. Speech recordings (picture description) and self-report questionnaires are collected directly after task execution. The colors used in the figure are congruent with the colors presented in the results section.

The experiment was designed in OpenSesame version 3.2.8 and was carried out on a dedicated computer (Dell, Windows 10). Participants came in on two different days, at least 7 days apart. The experimental sessions were identically structured, but only the task contents differed (Day1; see Cyberball, Day2; see MIST). Prior to the task, electrodes (ECG, EDA; see Physiological Data) and an EEG cap were placed (duration 10–30 min; beyond this paper’s scope). The experiment started with a 10-min (5 min eyes closed, 5 min eyes open) resting block to achieve habituation. After this, the Control task started. After this condition, there was another 10-min resting block, followed by a Stress task and another 10-min resting block. Subsequent to each task block (i.e., Control task and Stress task), participants were prompted to do a speech trial (see Speech Data) and respond to self-report psychological state questionnaires (see Self-Report Data). See Fig. 2 for a flowchart figure.

#### Cyberball—Day 1

The Cyberball paradigm involved a ball-tossing game in which participants played with two computer-generated confederates (one man, one woman, placement counterbalanced across participants), represented by pictures (from Allaert and colleagues^29^). However, participants were told that the other players were humans participating at other universities. The confederates' behaviors were predefined and the game was visualized with a picture of the participant at the bottom center, while the confederate pictures were placed at the top left and right^19^. Participants could throw the ball to either confederate by pressing an arrow key (right hand), and the ball's movement took 1500 ms. The confederates held possession between two and three seconds (randomly generated) to increase the credibility that they were human. During the **control task** (inclusion phase), participants received the ball 33% of the time over 150 throws, while in the **stress task** (exclusion phase), participants were excluded in a probabilistic manner from receiving the ball after an initial normal phase of 30 throws. The chance of retrieving the ball increased with each subsequent throw not directed at the participant, with chances ranging from 0 to 100% (adapted from Williams and colleagues^19^).

#### Montreal Imaging Stress Task (MIST)—Day 2

During the Montreal Imaging Stress Task (MIST), participants solved mathematical equations of increasing difficulty^20^. Equations were displayed in black on a white background, and the correct answer was always a number between zero and nine, with participants answering each question using the corresponding number on the keyboard's numpad (right hand). The difficulty scales and equation-generating code was identical to the original study, as supplied by prof. Pruessner^20^.

The **control task** included seven difficulty scales, with participants solving up to ten equations per scale. After each equation, feedback was given in the form of *"Correct!"*, *"Incorrect!"*, or *"Timeout!",* shown in black. The **stress task** employed the same difficulty scales but introduced changes to the task and feedback. Participants were informed that their performance would be compared to that of a group and that they should perform at least on par with the average. Equations were presented with a shrinking bar indicating the remaining time to solve the equation, and the allowed time was set to be 90% of their average response time during the control task. After every three successive correct or incorrect answers, the allowed time was adjusted by 10% to increase/decrease difficulty. Participants also saw a performance bar with two arrows indicating their personal and group average scores. Their personal arrow moved in steps of 5% of the bar’s length after each equation (incorrect/timeout; left, correct; right), whereas the group average arrow was stationary at 83%. If a participant’s performance fell below the average group performance after completing five difficulty scales, the experimenter would inform them that their data might not be usable and urge them to improve.

### Data collection

In this study, several types of data were collected for analysis. While our main hypotheses focused on specific data modalities, we also collected additional self-report and cardiac data. To ensure transparency, we have provided an overview of these data modalities and a complete study flowchart in the supplemental materials.

#### Speech data

On both days, after completion of either task (i.e., control/stress task), participants were prompted to describe a picture out loud, see Fig. 2. The image was a screenshot of the task they had just completed to avoid introducing noise to our self-report measures by having their minds wander (for a similar approach and considerations, see Van Der Donckt and colleagues^13^). The participants were instructed to describe the images based on what they saw, as well as how it made them feel. See supplemental materials for screenshots.

#### Self-report data

On both days, after completion of either task (i.e., control/stress task), participants were asked to rate their current levels of stress and negative affect (“Right now, how much do you feel…”) using 6 negative affect (NA) prompts and 1 stress prompt question, each with a 0–100 sliding scale (0 = Not at all—100 = Very much), see Fig. 2. The six negative affect (NA) prompts are: upset, distressed, scared, angry, anxious, and sad, whereas stress was a single item asking “Right now, how much do you feel stressed?”. Positive activating and soothing affect were also collected but not part of the primary hypotheses, thus only described in the supplemental materials. These scales were adopted from Petrocchi and colleagues^30^. Given the high internal consistency between the prompts in the NA category, these responses were aggregated to compute a single mean score to be used in the analysis.

#### Physiological data

We collected electrocardiography (ECG; see supplemental material for more info) and electrodermal activity (EDA) data throughout the paradigms using the VU-AMS ambulatory monitor (VU University Amsterdam, www.vu-ams.nl, Amsterdam, the Netherlands), which was specifically designed for this purpose. To collect EDA data, we placed two velcro electrodes with isotonic electrode gel (Biopac) on the middle phalanges of the left index and middle finger.

### Data analysis

#### Physiological data

EDA data was preprocessed using specific Python code, which can be found in the supplemental material (0.2_EDA.ipynb & scl_processing.py). To prepare the data for analysis, a 2 Hz low-pass filter was applied to the raw signal, which was then decomposed into a tonic and phasic component. From the phasic component, the Skin Conductance Response Rate (SCRR) was extracted by identifying peaks with the SciPy toolkit. The thresholds for rise and fall time, as well as peak parameters, were determined based on established guidelines from the literature^31^.

#### Extraction of speech features

To ensure data quality, we manually checked all recordings whether they were complete, clear, with limited background noise, and no excessive clipping. In addition, recordings were dropped if there was no complementary self-report scales (due to technical issues). Eight recordings were removed, resulting in 120 control recordings (64 Cyberball, 56 MIST) of 66 out of 66 participants, and 119 stress recordings (64 Cyberball, 55 MIST) of 66 out of 66 participants. Prior to feature extraction, we downsampled the speech samples to 16 kHz and applied dithering. These steps were performed in order to make the extracted OpenSMILE metrics less sensitive to environmental harmonics at the voiced boundaries^13^. To extract features from the recordings, we used OpenSmile 2.3.0^32^ with the GeMAPSv01b configuration^33^, a widely-used acoustic feature set in voice research and affective computing. From this feature set (feature names as described in GeMAPS added between brackets), we selected Fundamental Frequency (F0; F0semitoneFrom27.5Hz_sma3nz_amean), Jitter (jitterLocal_sma3nz_amean), Shimmer (shimmerLocaldB_sma3nz_amean), Harmonics-to-Noise Ratio (HNR; HNRdBACF_sma3nz_amean), and Voiced Segment Length (MeanVoicedSegmentLengthSec) and Mean Voiced Segments per Second (a proxy for speech speed; VoicedSegmentsPerSec) to capture changes in speech rate. All features were computed using Python 3.9.6 for a sliding window and then mean-aggregated over the whole recording, thus not displaying high temporal changes. For detailed information regarding feature calculation and extraction procedure, we refer the reader to Eyben et al.^32^ and Section 6.1 of Eyben et al.^33^.

#### Statistical analysis

Statistical analyses were performed using R4.1.1 (for detailed version information of the software and packages used, please refer to the supplemental materials).

We used the ‘lme4′^34^ package to fit linear mixed models (LMMs) to each of the dependent variables. The sum of squares for each model was estimated using a partial sum of squares (Anova type III approach), and the statistical significance level was set to *p* < 0.05 (these results are only reported in the supplemental materials). Tests for pairwise comparisons of the EMMs (estimated marginal means) were performed with the ‘emmeans’ package^35^. A false discovery rate (FDR) was used to correct for multiple comparisons correction for each data modality (e.g., all speech comparisons pooled together and penalized accordingly) to minimize the risk of Type 1 errors^36^ using the ‘p.adjust()’ function from the ‘stats’ package. In the results section, only corrected p-values will be reported. Moreover, effect sizes (Cohen’s D) and their 95% confidence intervals (CI) are estimated with the ‘eff_size()’ function from the ‘emmeans’ package^35^. Results are only reported using the effect sizes of within-paradigm comparisons to make a comparison between different dependent variables and data modalities. Note that between-paradigm comparisons (i.e., Cyberbal vs MIST) should be avoided for each respective task (i.e., control and stress task) as the performed tasks and the described pictures are inherently different.

To control for the potential effect of gender on the different dependent variables, gender was considered as a fixed effect for each individual model prior to statistical inference. However, to make sure our models were parsimonious, we bottom-up tested whether adding *gender* as an independent variable to the model improved each model’s fit^37^. For each dependent variable, we compared models that included and excluded *gender*, and it was only included in the model if it showed to be a significant contributor after comparing models with reducing complexity using χ^2^ goodness-of-fit tests within the ‘anova()’ function^38^. The statistical significance level was set to *p* < 0.05 and based on this, gender was included in the models for F0, Shimmer, HNR, Voiced segment length, and self-reported stress. As such, each model followed the following structure; *DependentVariable* ~ Phase * Task + *Gender* + *(1|ID)* or *DependentVariable* ~ Phase * Task + *(1|ID)*. With *Phase* having 2 levels (control vs stress), *Task* having 2 levels (Cyberball vs MIST), and participant as a random intercept.

### Supplementary Information

Supplementary Information.

## Data Availability

All data and corresponding code are openly available at https://osf.io/qf6ck/https://github.com/mitchelkappen/stress_cyberball-mist.
