# Supplementary material for: Acoustic and prosodic speech features reflect physiological stress but not isolated negative affect: a multi-paradigm study on psychosocial stressors
Source: Sci Rep. 2024 Mar 6;14:5515. doi: 10.1038/s41598-024-55550-3 (PMC10918109; doi:10.1038/s41598-024-55550-3)
Supplement: Supplementary file 1 — Supplementary Information. [file 41598_2024_55550_MOESM1_ESM.docx]

Supplementary Materials

For

*Acoustic and Prosodic Speech Features Reflect Physiological Stress but not Isolated Negative Affect: A Multi-Paradigm Study on Psychosocial Stressors*

Mitchel Kappen, Gert Vanhollebeke, Jonas Van Der Donckt,

Sofie Van Hoecke, Marie-Anne Vanderhasselt

## Open Science Statement

All data and corresponding code are openly available through Github and OSF. Code works out-of-the-box with instructions found in the corresponding README.md in OSF directory.

Github: <https://github.com/mitchelkappen/stress_cyberball-mist>

OSF: <https://osf.io/qf6ck/>

## Contents:

1. Exclusion Criteria
2. Complete study flowchart
3. Speech data collection screenshots
4. Self-reports

4.1) Positive activating affect

4.2) Positive soothing affect

1. ECG/HRV data
2. Software and packages used

6.1) R

6.2) Python

1. Full models & Anova results

7.1) Skin Conductance Response Rate (SCRR)

7.2) Negative Affect

7.3) Self-Reported Stress

7.4) Fundamental Frequency (F0)

7.5) Voiced segments per second

7.6) Voiced segment length

7.7) Harmonics-to-noise ratio (HNR)

7.8) Shimmer

7.9) Jitter

7.10) Correlation Table

## 1) Exclusion Criteria

Participants were not allowed to enroll in the study if they met any of the following criteria. Some of the criteria were directly related to the collection of EEG:

- Left-handed
- Born before 1977
- Born after 2003
- Personal or family history of epilepsy
- Recent neurosurgical procedures
- Pacemaker or other electronic implants
- Inner ear prosthesis
- Metal objects or magnetic objects in the brain or around the head (only removable earrings & piercings are allowed)
- Pregnancy
- A current depressive episode
- Other psychiatric disorders
- Skin condition on the head
- Current addiction
- Current substance abuse
- Current use of psychotropic medication
- Eye disease(s)
- Heart, respiratory, or neurological problems
- Participated in the EEG study "predicting future success"
- Psychology students
- Dreadlocks
- Tightly curled hair

## 2) Complete study flowchart


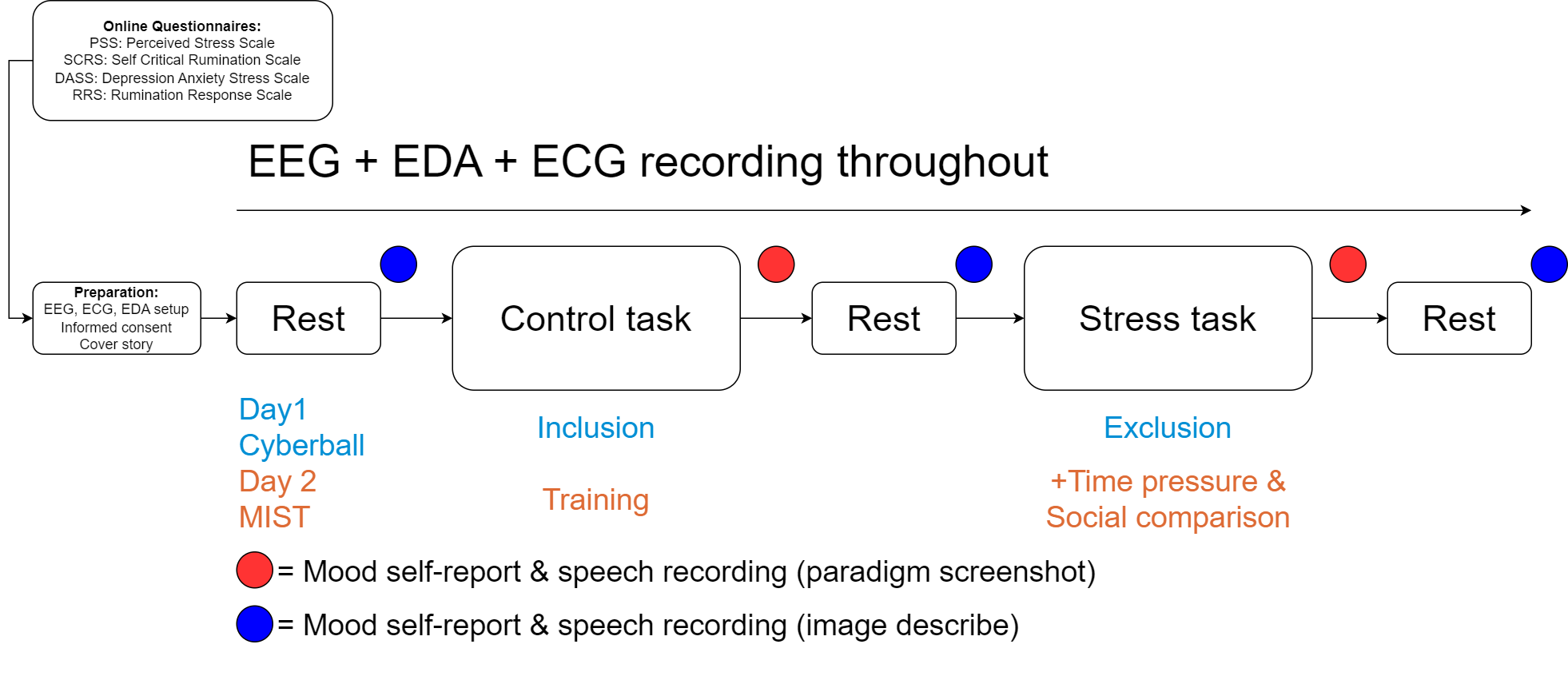


##

## 3) Speech data collection screenshots

All speech trials were preceded by the following screen:


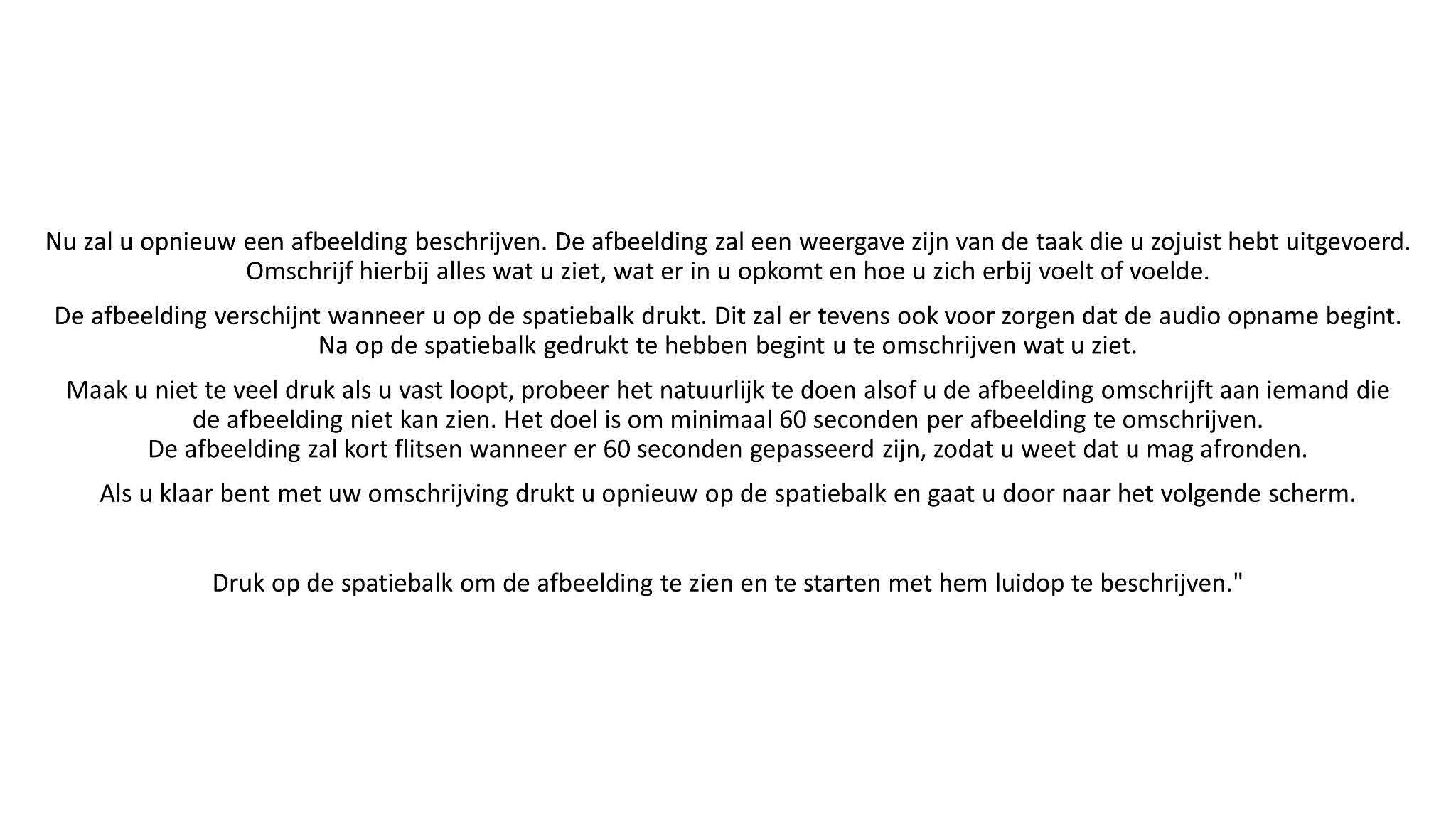


**Translation in English:**

*“Now you will describe an image again. The image will be a representation of the task you just performed. Describe everything you see, what comes to mind, and how you feel or felt about it.*

*The image appears when you press the spacebar. This will also start the audio recording. After pressing the spacebar, start describing what you see.*

*Don't worry too much if you get stuck, try to do it naturally as if you are describing the image to someone who cannot see it. The goal is to describe for at least 60 seconds per image. The image will briefly flash when 60 seconds have passed so that you know you can finish up.*

*When you have finished your description, press the spacebar again and proceed to the next screen.*

*Press the spacebar to see the image and start describing it aloud.”*

All screenshot description trials looked identical per participant. After describing for 60 seconds, a prompt appeared saying “You can now press the space bar to stop the audio recording”.

## Cyberball

**Inclusion**:


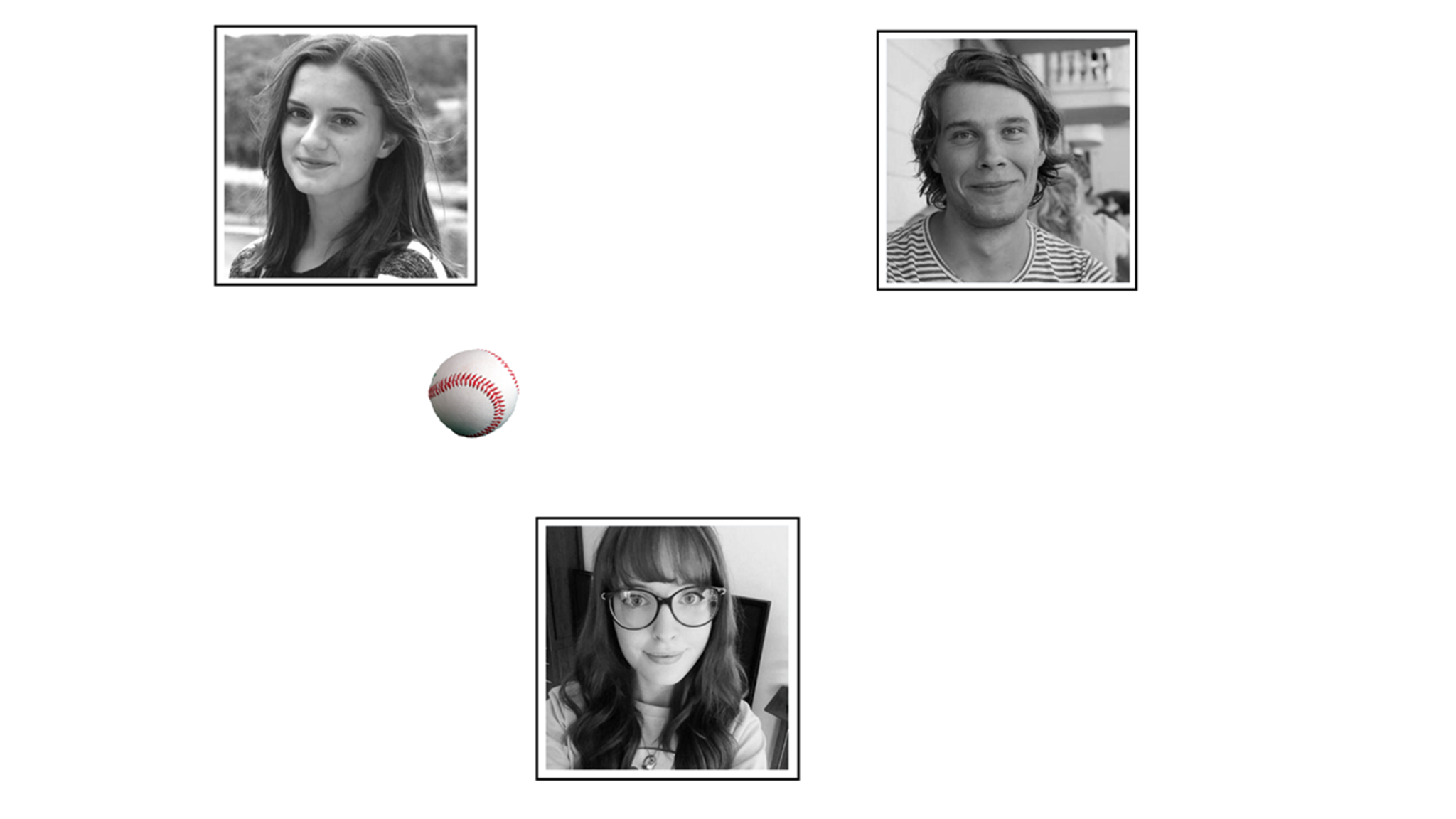


**Exclusion:**


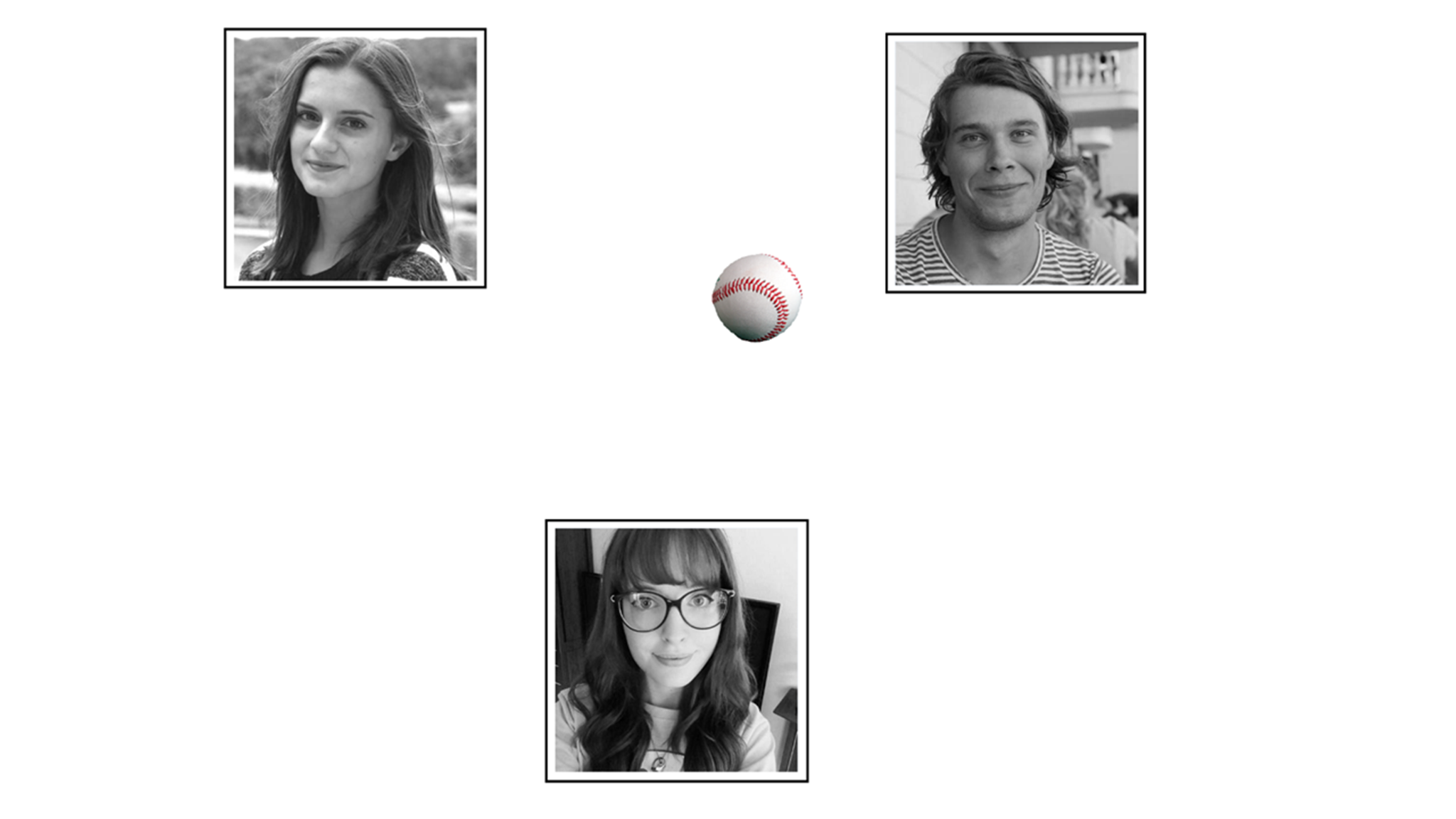


## MIST

**Control**:


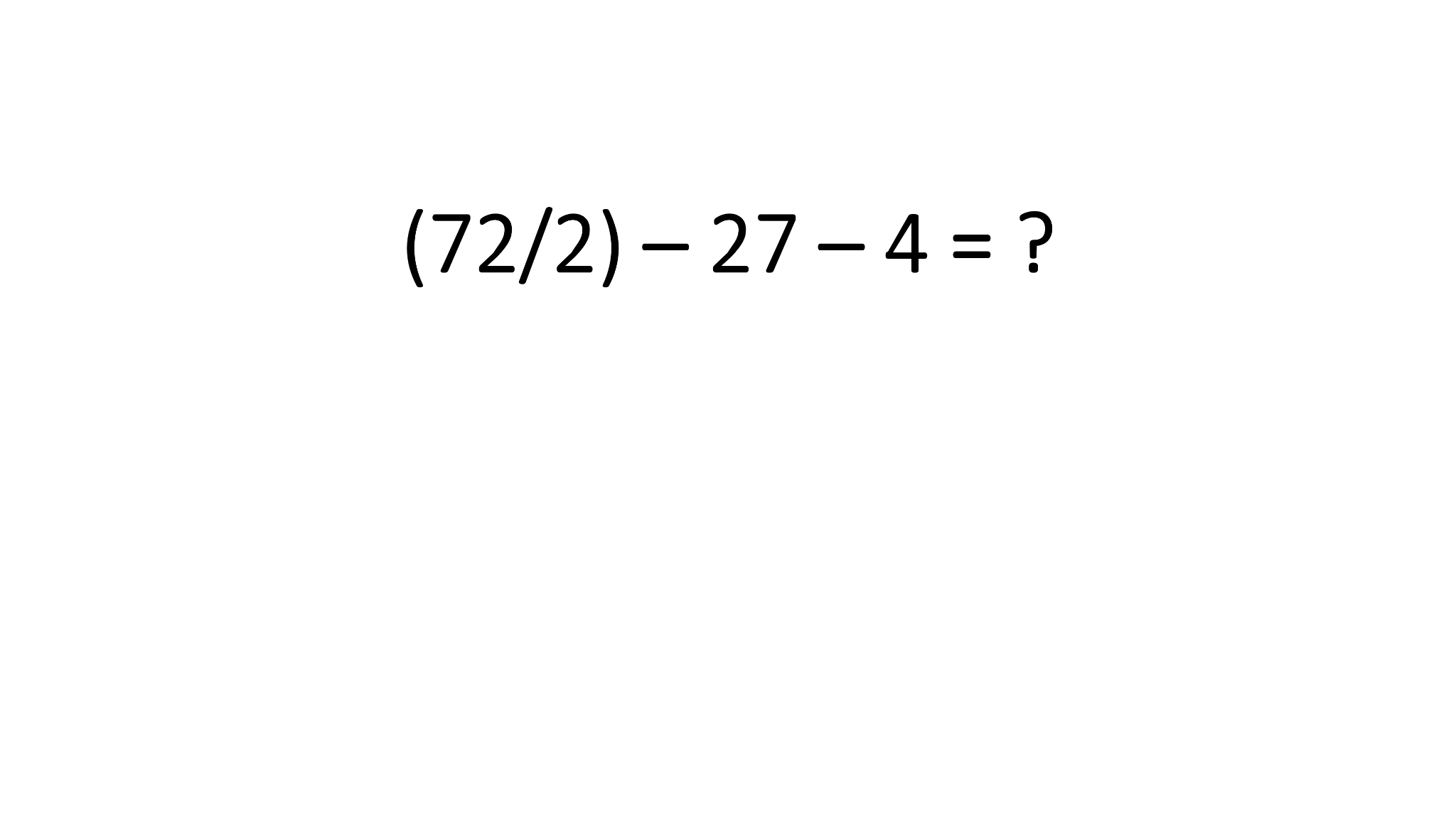


**Stress**:


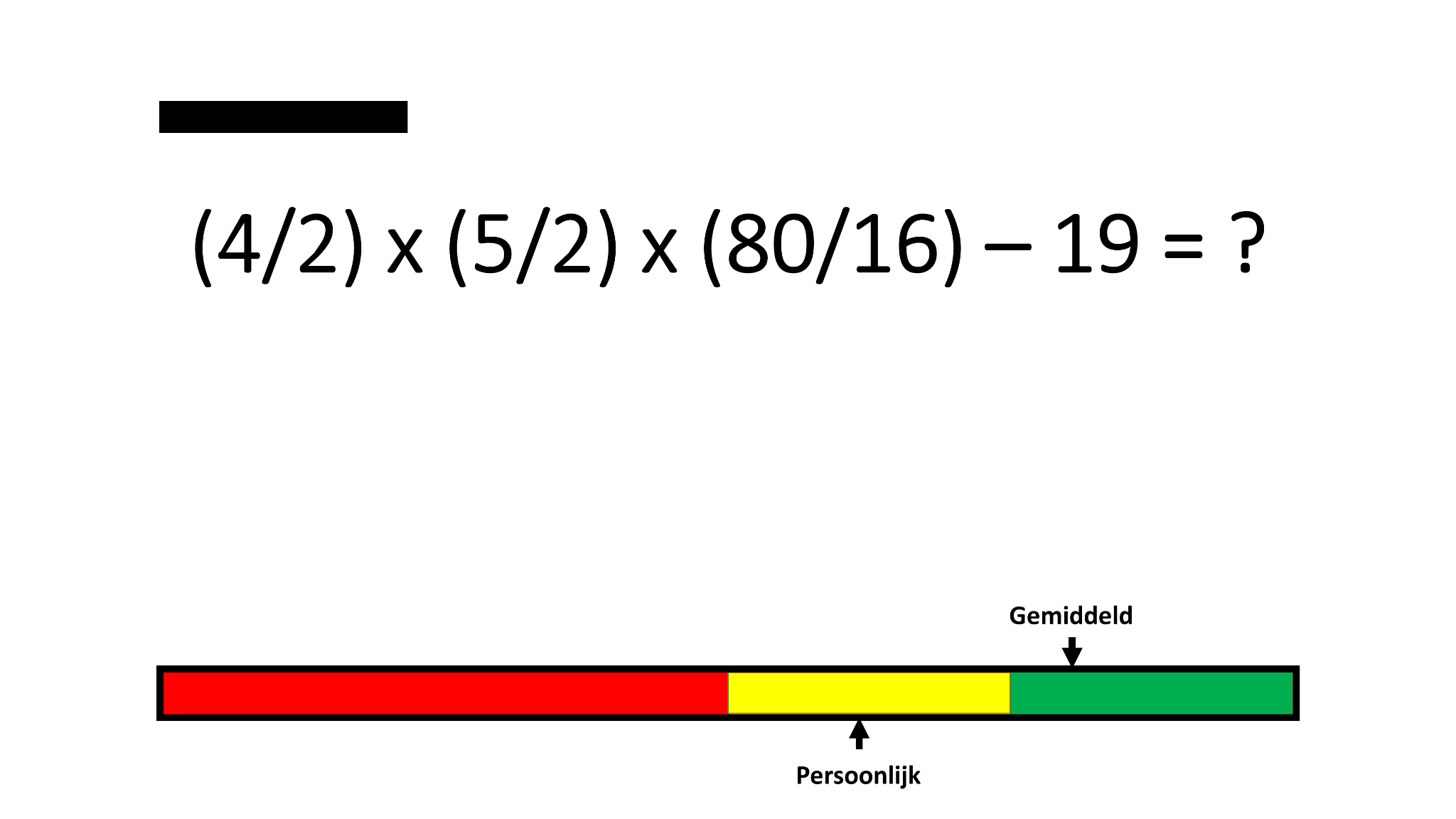


## 4) Self-reports

## 4.1) Positive activating affect

**Anova:**

Analysis of Deviance Table (Type III Wald chisquare tests)

Response: VAS_PAA

Chisq Df Pr(>Chisq)

(Intercept) 318.0398 1 < 2.2e-16 ***

fileNum 12.6142 1 0.0003828 ***

taskType 1.7137 1 0.1905012

fileNum:taskType 12.5367 1 0.0003990 ***

---

Signif. codes: 0 ‘***’ 0.001 ‘**’ 0.01 ‘*’ 0.05 ‘.’ 0.1 ‘ ’ 1

**Emmeans Contrasts:**

taskType = Cyberball:

contrast estimate SE df t.ratio p.value

Control Task - Stress Task 11.672 2.24 170 5.207 <.0001

taskType = MIST:

contrast estimate SE df t.ratio p.value

Control Task - Stress Task 0.018 2.41 170 0.007 0.9941

Degrees-of-freedom method: kenward-roger

**Effect Sizes:**

taskType = Cyberball:

contrast effect.size SE df lower.CL upper.CL

(Control Task - Stress Task) 0.92052 0.182 170 0.562 1.279

taskType = MIST:

contrast effect.size SE df lower.CL upper.CL

(Control Task - Stress Task) 0.00142 0.190 170 -0.374 0.377

sigma used for effect sizes: 12.68

Degrees-of-freedom method: inherited from kenward-roger when re-gridding

Confidence level used: 0.95

**Figure:**


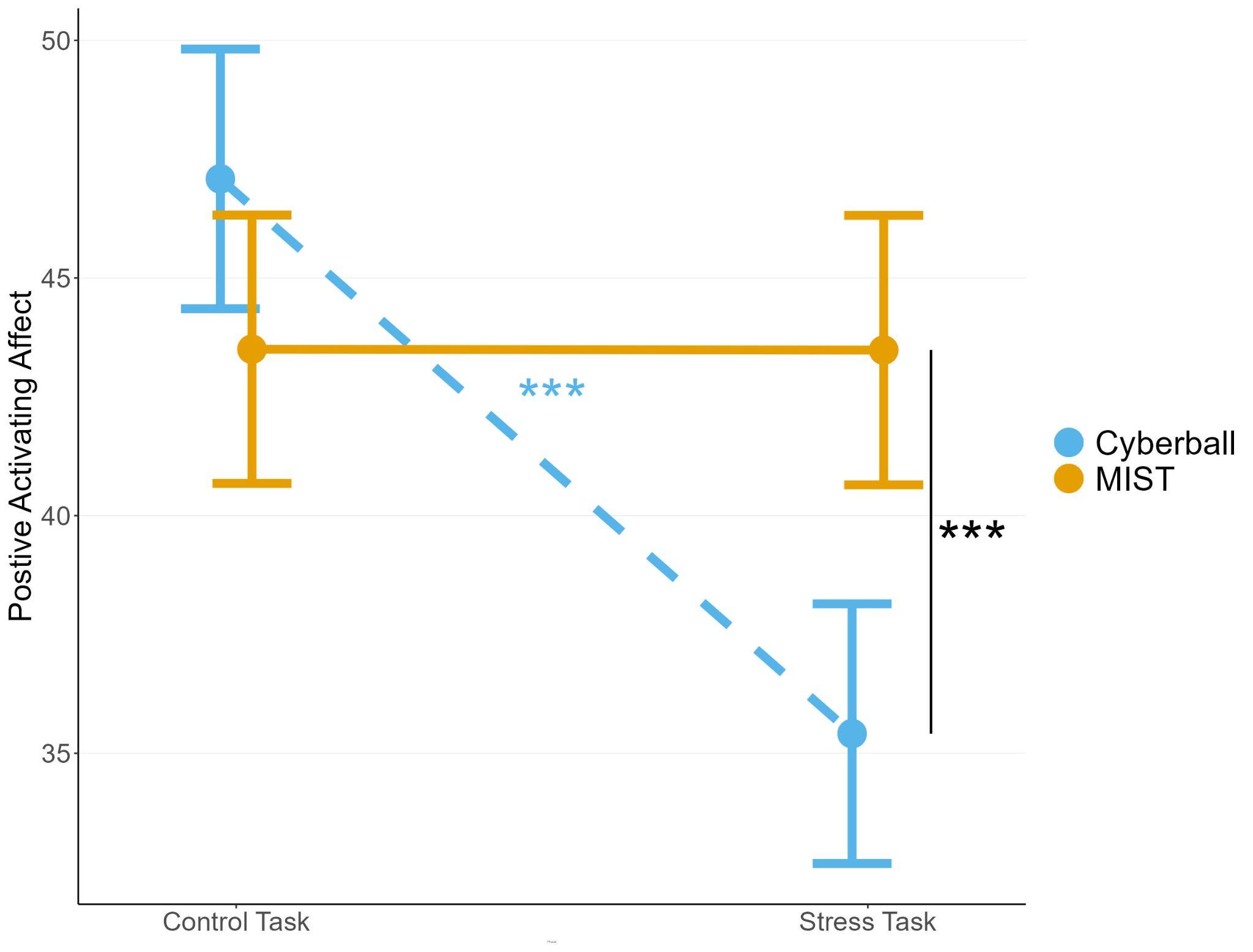


## 4.2) Positive soothing affect

**Anova:**

Analysis of Deviance Table (Type III Wald chisquare tests)

Response: VAS_PSA

Chisq Df Pr(>Chisq)

(Intercept) 925.4036 1 < 2.2e-16 ***

fileNum 35.1251 1 3.092e-09 ***

taskType 73.6405 1 < 2.2e-16 ***

fileNum:taskType 1.5812 1 0.2086

---

Signif. codes: 0 ‘***’ 0.001 ‘**’ 0.01 ‘*’ 0.05 ‘.’ 0.1 ‘ ’ 1

**Emmeans Contrasts:**

taskType = Cyberball:

contrast estimate SE df t.ratio p.value

Control Task - Stress Task 9.42 2.75 170 3.428 0.0008

taskType = MIST:

contrast estimate SE df t.ratio p.value

Control Task - Stress Task 14.50 2.96 171 4.906 <.0001

Degrees-of-freedom method: kenward-roger

**Effect Sizes:**

taskType = Cyberball:

contrast effect.size SE df lower.CL upper.CL

(Control Task - Stress Task) 0.606 0.179 170 0.253 0.959

taskType = MIST:

contrast effect.size SE df lower.CL upper.CL

(Control Task - Stress Task) 0.932 0.195 171 0.548 1.317

sigma used for effect sizes: 15.55

Degrees-of-freedom method: inherited from kenward-roger when re-gridding

Confidence level used: 0.95

**Figure:**


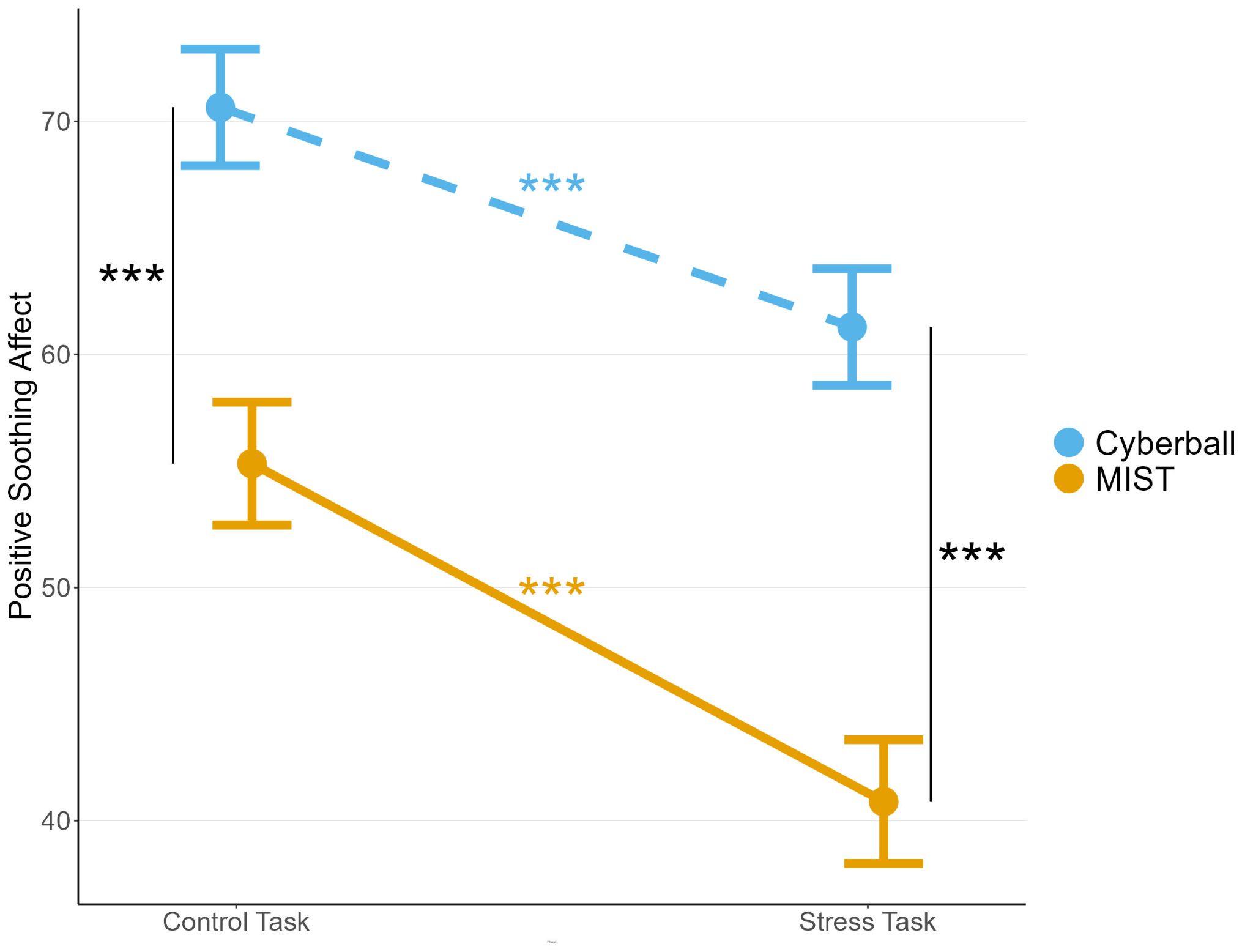


## 5) ECG/HRV data

**Anova:**

Analysis of Deviance Table (Type III Wald chisquare tests)

Response: rmssd

Chisq Df Pr(>Chisq)

(Intercept) 181.7315 1 <2e-16 ***

fileNum 2.7029 1 0.1002

taskType 0.9538 1 0.3288

fileNum:taskType 0.0224 1 0.8810

---

Signif. codes: 0 ‘***’ 0.001 ‘**’ 0.01 ‘*’ 0.05 ‘.’ 0.1 ‘ ’ 1

**Emmeans Contrasts:**

taskType = Cyberball:

contrast estimate SE df t.ratio p.value

Control Task - Stress Task -3.78 2.90 158 -1.302 0.1947

taskType = MIST:

contrast estimate SE df t.ratio p.value

Control Task - Stress Task -3.15 3.05 158 -1.034 0.3028

Degrees-of-freedom method: kenward-roger

**Effect Sizes:**

taskType = Cyberball:

contrast effect.size SE df lower.CL upper.CL

(Control Task - Stress Task) -0.241 0.185 158 -0.607 0.125

taskType = MIST:

contrast effect.size SE df lower.CL upper.CL

(Control Task - Stress Task) -0.201 0.195 158 -0.585 0.183

sigma used for effect sizes: 15.68

Degrees-of-freedom method: inherited from kenward-roger when re-gridding

Confidence level used: 0.95

**Figure:**


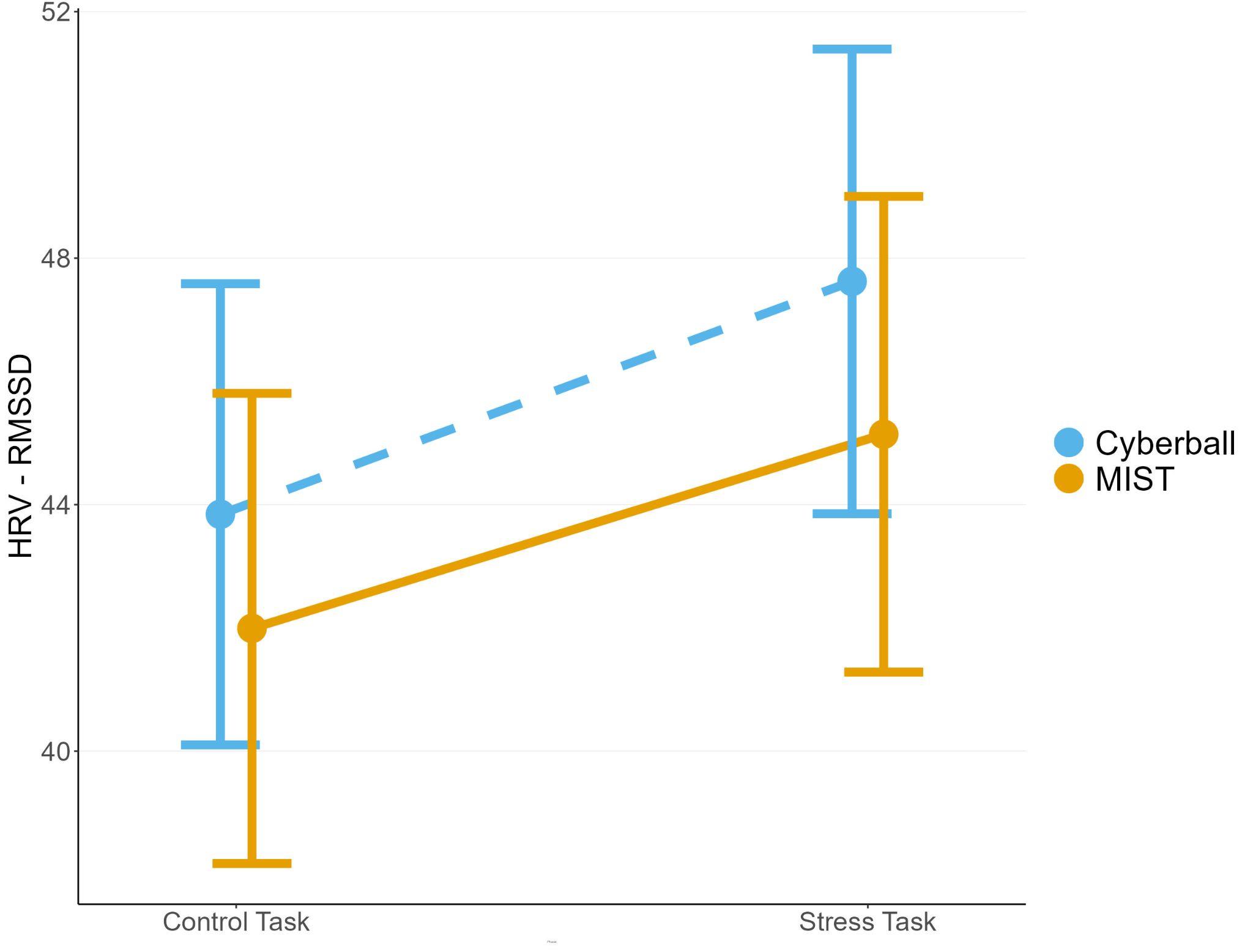


## 6) Software and packages used

For full package and version info see:

## 6.1) R

<https://github.com/mitchelkappen/stress_cyberball-mist/blob/main/supplemental%20material/package%20and%20version%20info/Rsession_info.txt>

## 6.2) Python

<https://github.com/mitchelkappen/stress_cyberball-mist/blob/main/supplemental%20material/package%20and%20version%20info/poetry.lock>

##

## 7) Full models & Anova results

Here you will find each model specification with the Anova results. For more details, feel free to run our out-of-the-box code in allAnalysis.R

We display all comparisons, both within-paradigm as between-paradigm for completeness purposes. However, it should be noted that no weight should be given to the between-paradigm comparisons due to the inherent differences in the paradigms and the images that were described.

## 7.1) Skin Conductance Response Rate (SCRR)

**Formula:** SCRR ~ fileNum * taskType + (1|participantNum)

**Anova:**

Analysis of Deviance Table (Type III Wald chisquare tests)

Response: SCRR

Chisq Df Pr(>Chisq)

(Intercept) 194.5865 1 < 2.2e-16 ***

fileNum 15.1337 1 0.0001002 ***

taskType 94.3411 1 < 2.2e-16 ***

fileNum:taskType 2.6339 1 0.1046055

---

Signif. codes: 0 ‘***’ 0.001 ‘**’ 0.01 ‘*’ 0.05 ‘.’ 0.1 ‘ ’ 1

**Figure:**

**
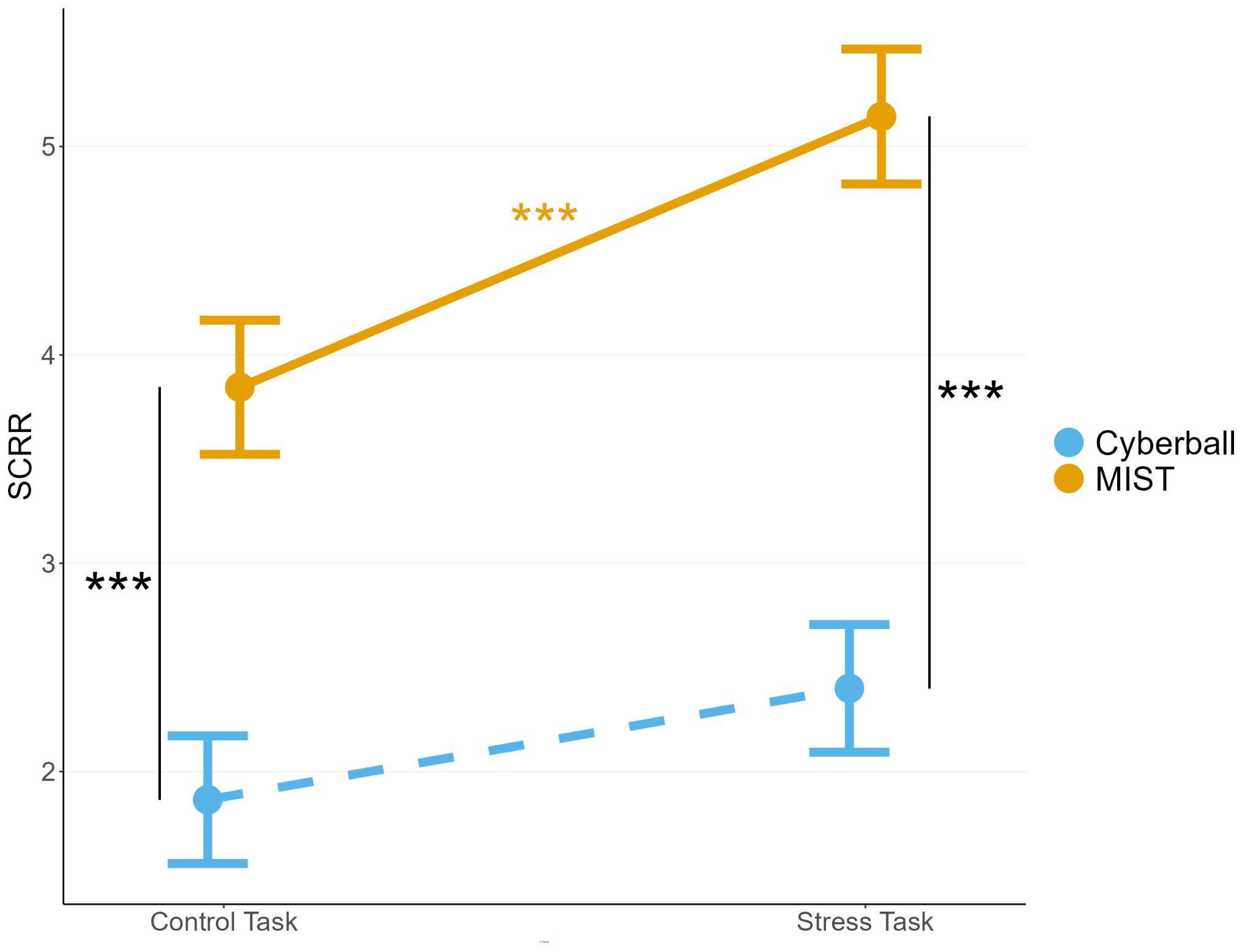
**

## 7.2) Negative Affect

**Formula:** VAS_NA ~ fileNum * taskType + (1|participantNum)

**Anova:**

Analysis of Deviance Table (Type III Wald chisquare tests)

Response: VAS_NA

Chisq Df Pr(>Chisq)

(Intercept) 63.7027 1 1.447e-15 ***

fileNum 17.5548 1 2.792e-05 ***

taskType 9.6001 1 0.001946 **

fileNum:taskType 0.2176 1 0.640868

---

Signif. codes: 0 ‘***’ 0.001 ‘**’ 0.01 ‘*’ 0.05 ‘.’ 0.1 ‘ ’ 1

**Figure:**


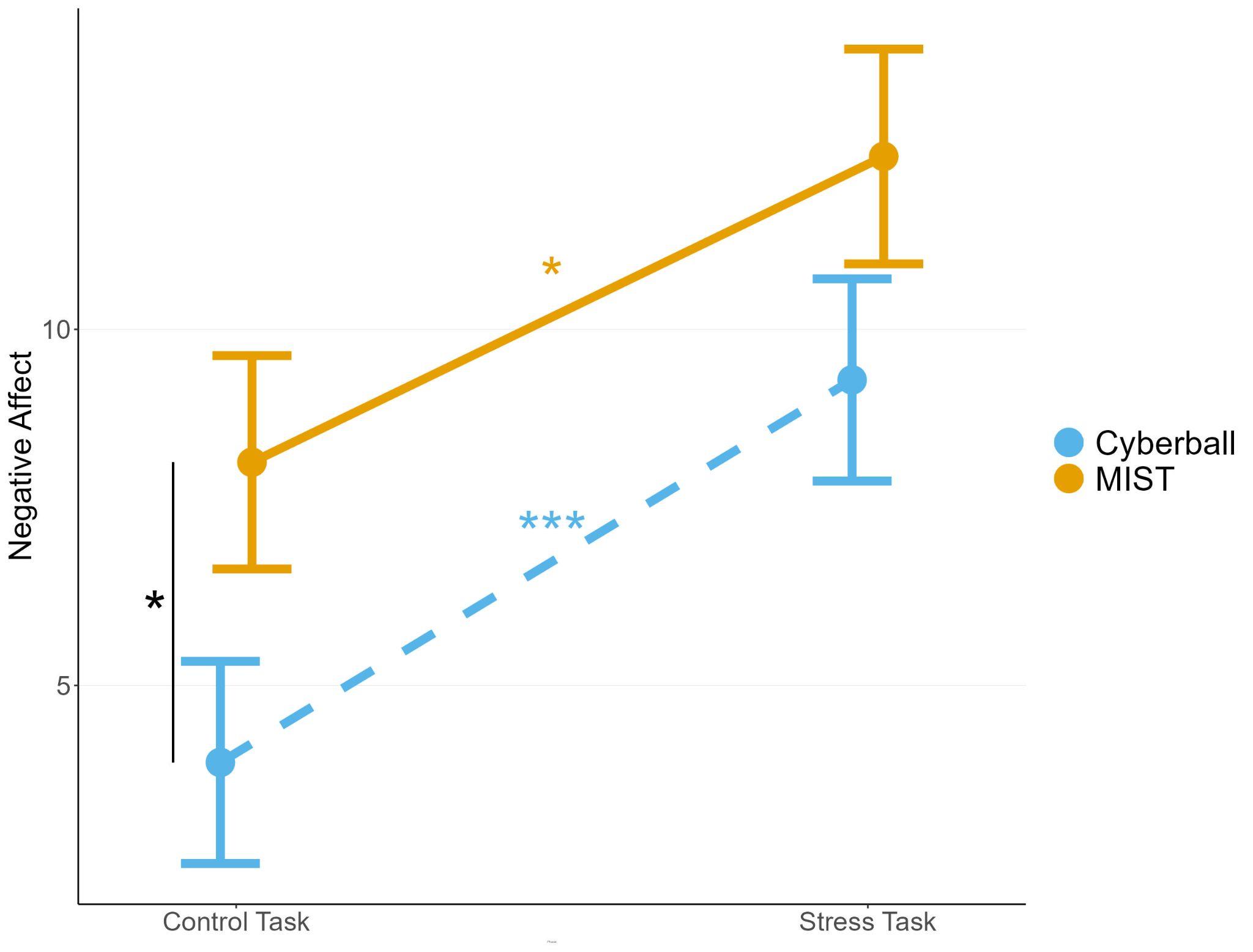


## 7.3) Self-Reported Stress

**Formula:** VAS_Stress ~ fileNum * taskType + Sex + (1|participantNum)

**Anova:**

Analysis of Deviance Table (Type III Wald chisquare tests)

Response: VAS_Stress

Chisq Df Pr(>Chisq)

(Intercept) 88.0639 1 < 2.2e-16 ***

fileNum 14.3127 1 0.0001548 ***

taskType 49.5210 1 1.963e-12 ***

Sex 7.7876 1 0.0052607 **

fileNum:taskType 7.8061 1 0.0052071 **

---

Signif. codes: 0 ‘***’ 0.001 ‘**’ 0.01 ‘*’ 0.05 ‘.’ 0.1 ‘ ’ 1

**Figure:**


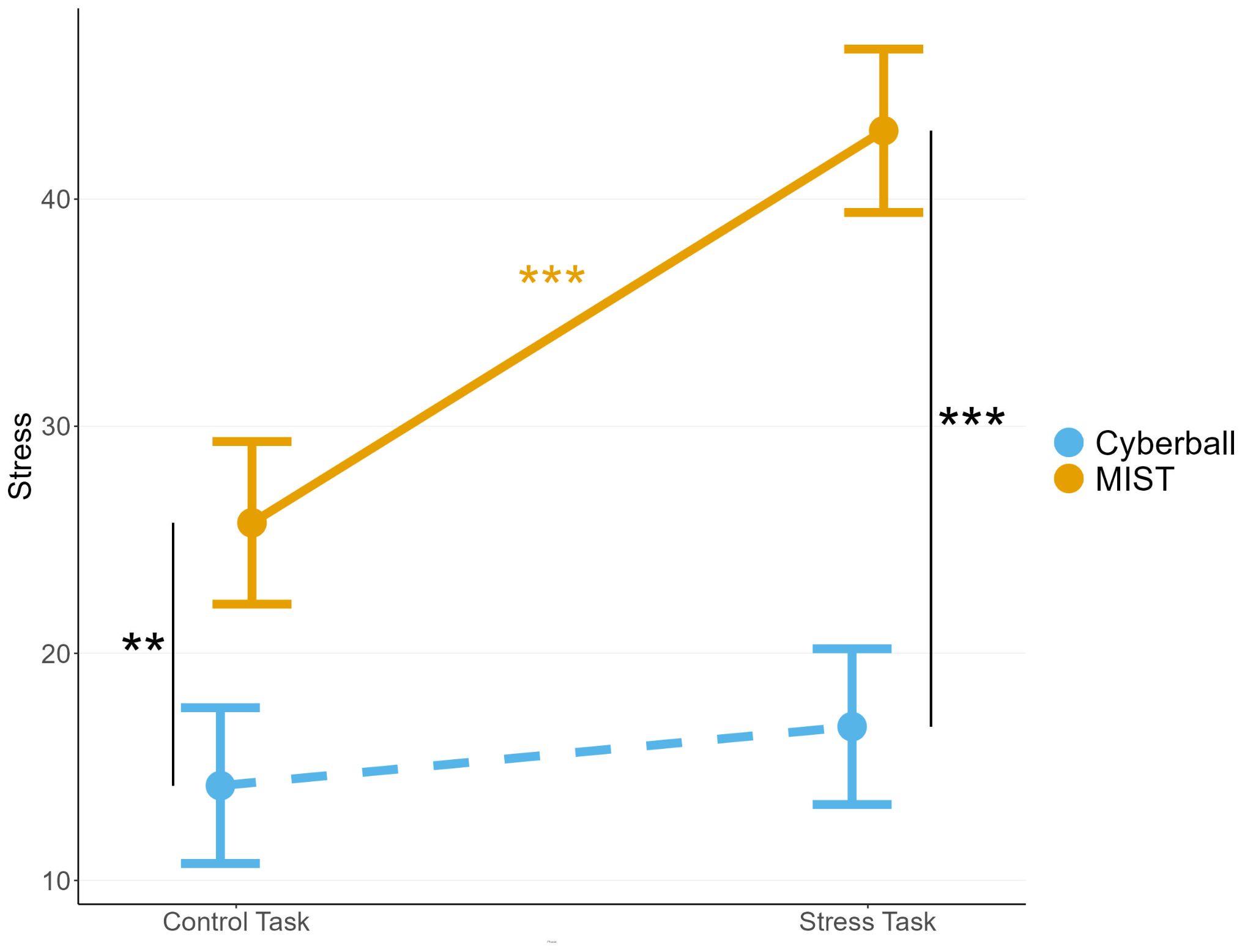


## 7.4) Fundamental Frequency (F0)

**Formula:** F0semitoneFrom27.5Hz_sma3nz_amean ~ fileNum * taskType + Sex + (1|participantNum)

**Anova:**

Analysis of Deviance Table (Type III Wald chisquare tests)

Response: F0semitoneFrom27.5Hz_sma3nz_amean

Chisq Df Pr(>Chisq)

(Intercept) 8108.1415 1 < 2.2e-16 ***

fileNum 6.7286 1 0.009488 **

taskType 0.0704 1 0.790737

Sex 196.7378 1 < 2.2e-16 ***

fileNum:taskType 2.0777 1 0.149464

---

Signif. codes: 0 ‘***’ 0.001 ‘**’ 0.01 ‘*’ 0.05 ‘.’ 0.1 ‘ ’ 1

**Figure:**


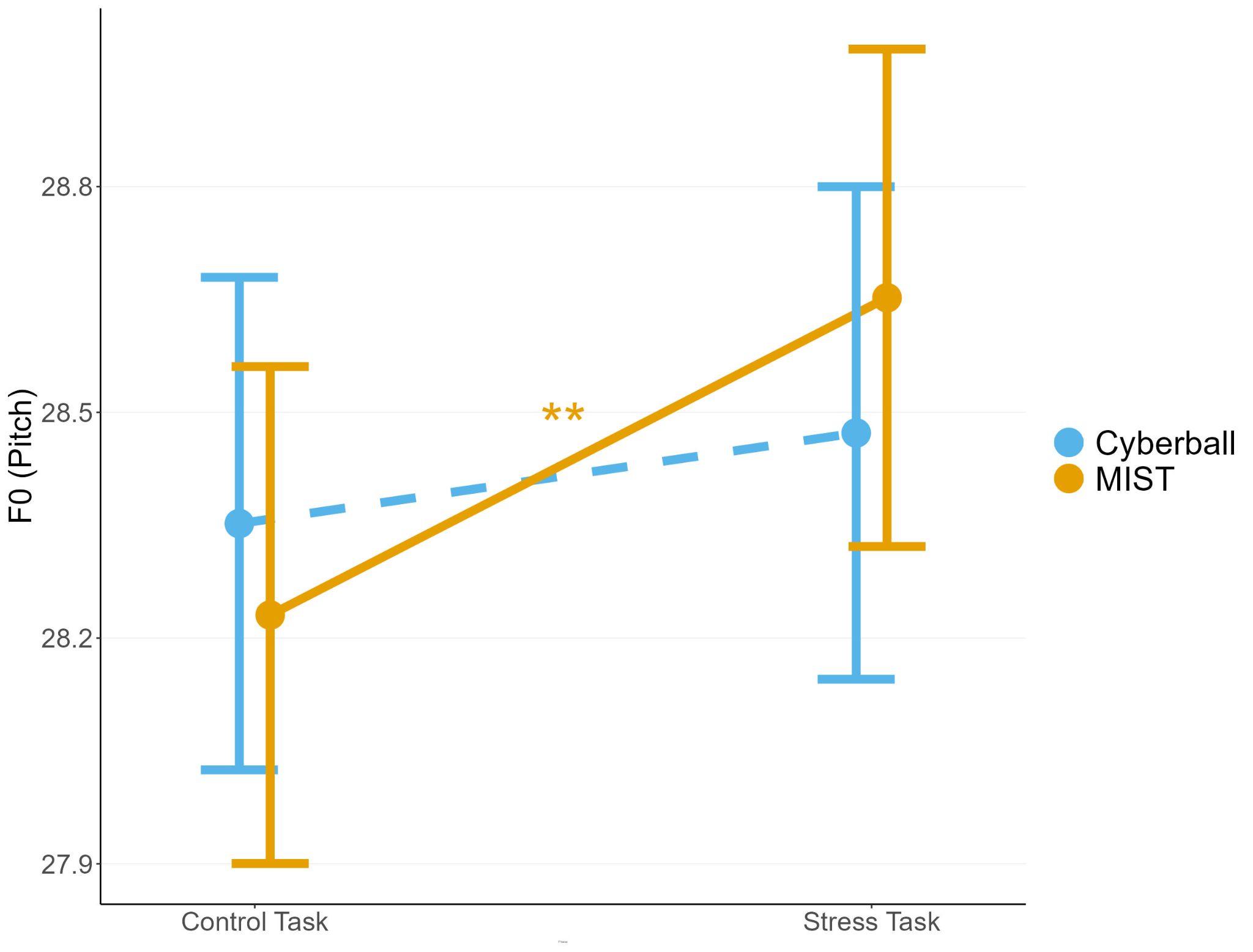


## 7.5) Voiced segments per second

**Formula:** VoicedSegmentsPerSec ~ fileNum * taskType + (1|participantNum)

**Anova:**

Analysis of Deviance Table (Type III Wald chisquare tests)

Response: VoicedSegmentsPerSec

Chisq Df Pr(>Chisq)

(Intercept) 2167.2753 1 < 2.2e-16 ***

fileNum 18.7850 1 1.463e-05 ***

taskType 0.6441 1 0.4222239

fileNum:taskType 13.1971 1 0.0002804 ***

---

Signif. codes: 0 ‘***’ 0.001 ‘**’ 0.01 ‘*’ 0.05 ‘.’ 0.1 ‘ ’ 1

**Figure:**


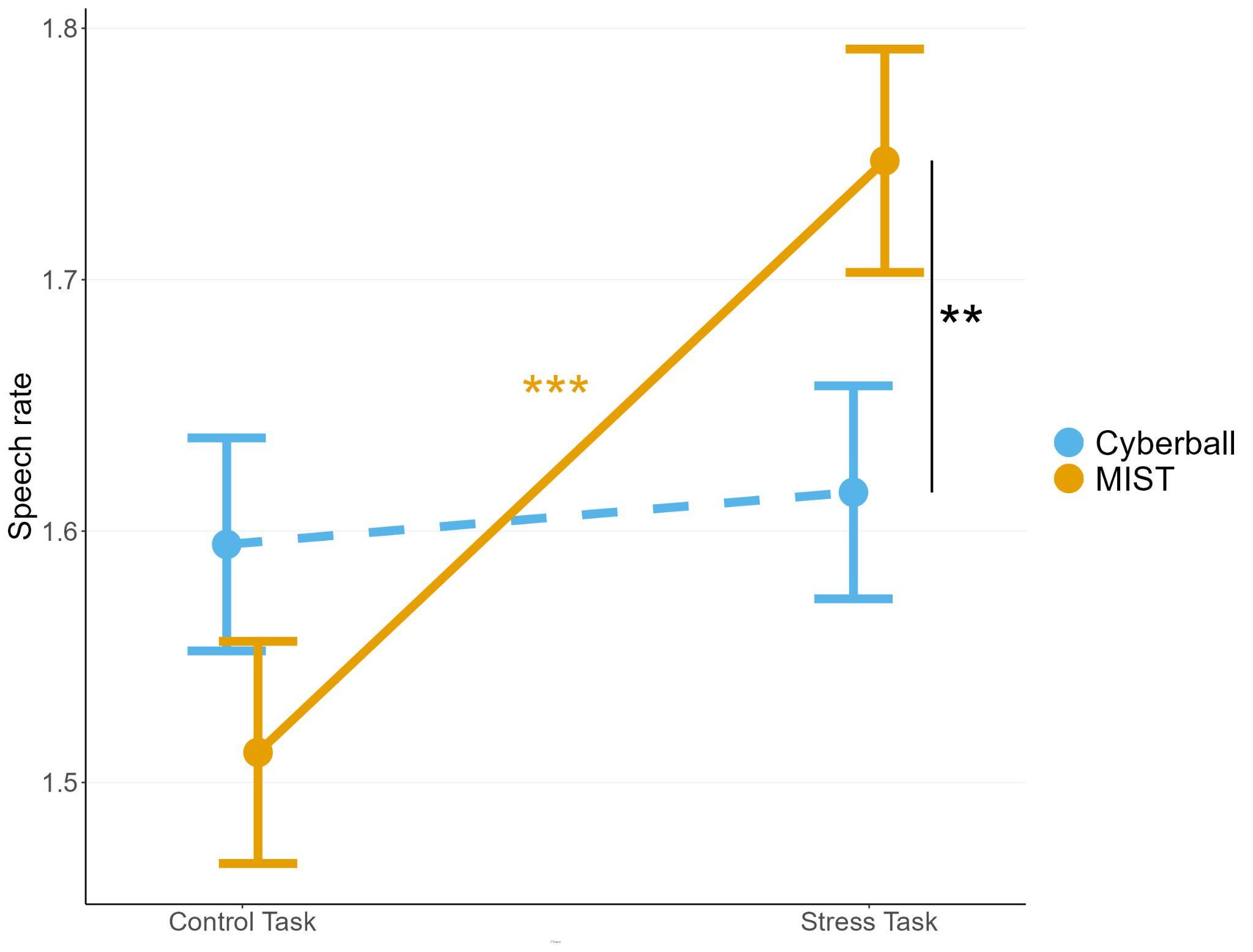


## 7.6) Voiced segment length

**Formula:** MeanVoicedSegmentLengthSec ~ fileNum * taskType + Sex + (1|participantNum)

**Anova:**

Analysis of Deviance Table (Type III Wald chisquare tests)

Response: MeanVoicedSegmentLengthSec

Chisq Df Pr(>Chisq)

(Intercept) 1237.4735 1 < 2e-16 ***

fileNum 3.0059 1 0.08296 .

taskType 76.3133 1 < 2e-16 ***

Sex 0.0049 1 0.94408

fileNum:taskType 4.4213 1 0.03549 *

---

Signif. codes: 0 ‘***’ 0.001 ‘**’ 0.01 ‘*’ 0.05 ‘.’ 0.1 ‘ ’ 1

**Figure:**


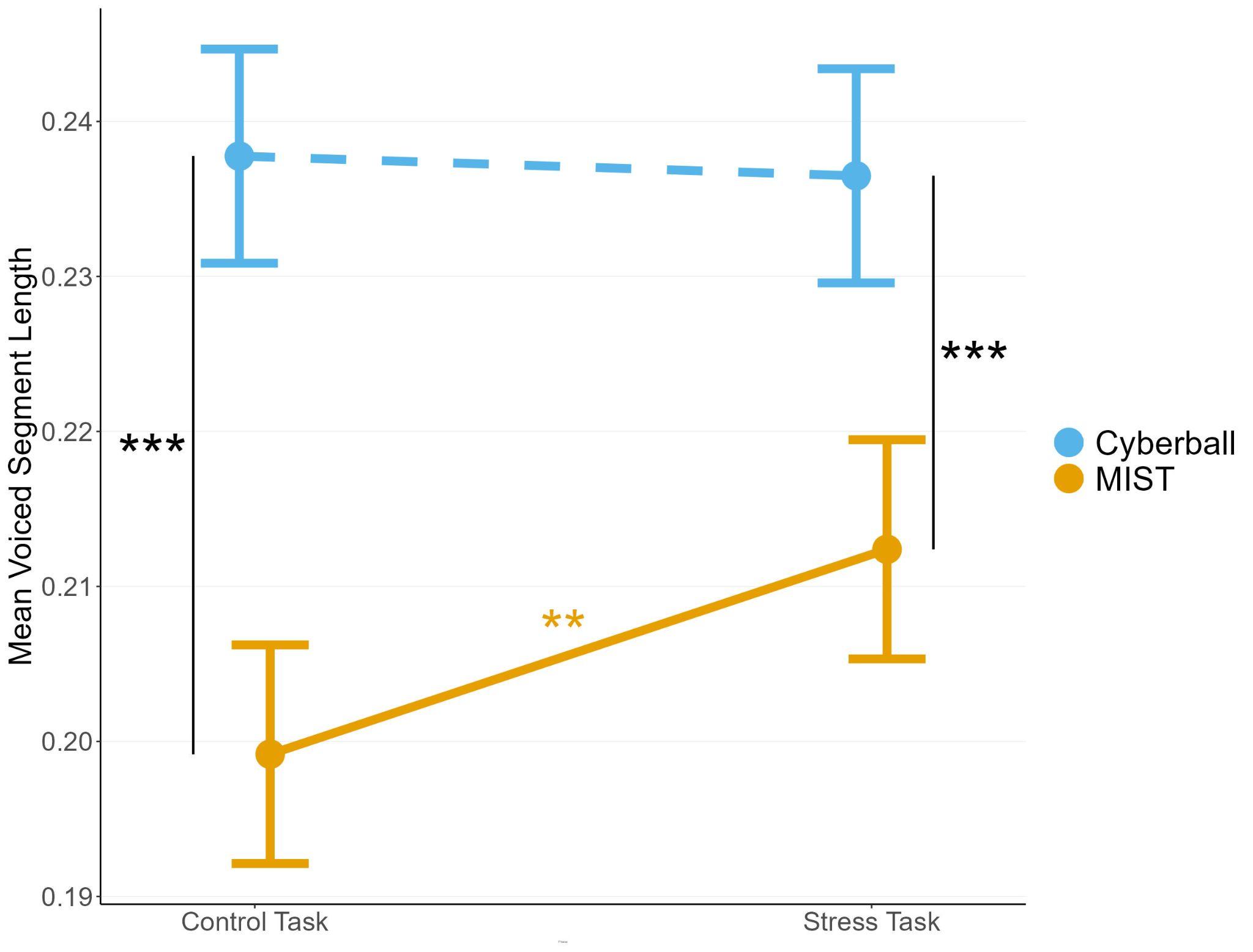


## 7.7) Harmonics-to-noise ratio (HNR)

**Formula:** HNRdBACF_sma3nz_amean ~ fileNum * taskType + Sex + (1|participantNum)

**Anova:**

Analysis of Deviance Table (Type III Wald chisquare tests)

Response: HNRdBACF_sma3nz_amean

Chisq Df Pr(>Chisq)

(Intercept) 2204.9603 1 < 2e-16 ***

fileNum 6.2719 1 0.01227 *

taskType 0.8847 1 0.34691

Sex 127.7845 1 < 2e-16 ***

fileNum:taskType 0.7817 1 0.37663

---

Signif. codes: 0 ‘***’ 0.001 ‘**’ 0.01 ‘*’ 0.05 ‘.’ 0.1 ‘ ’ 1

**Figure:**


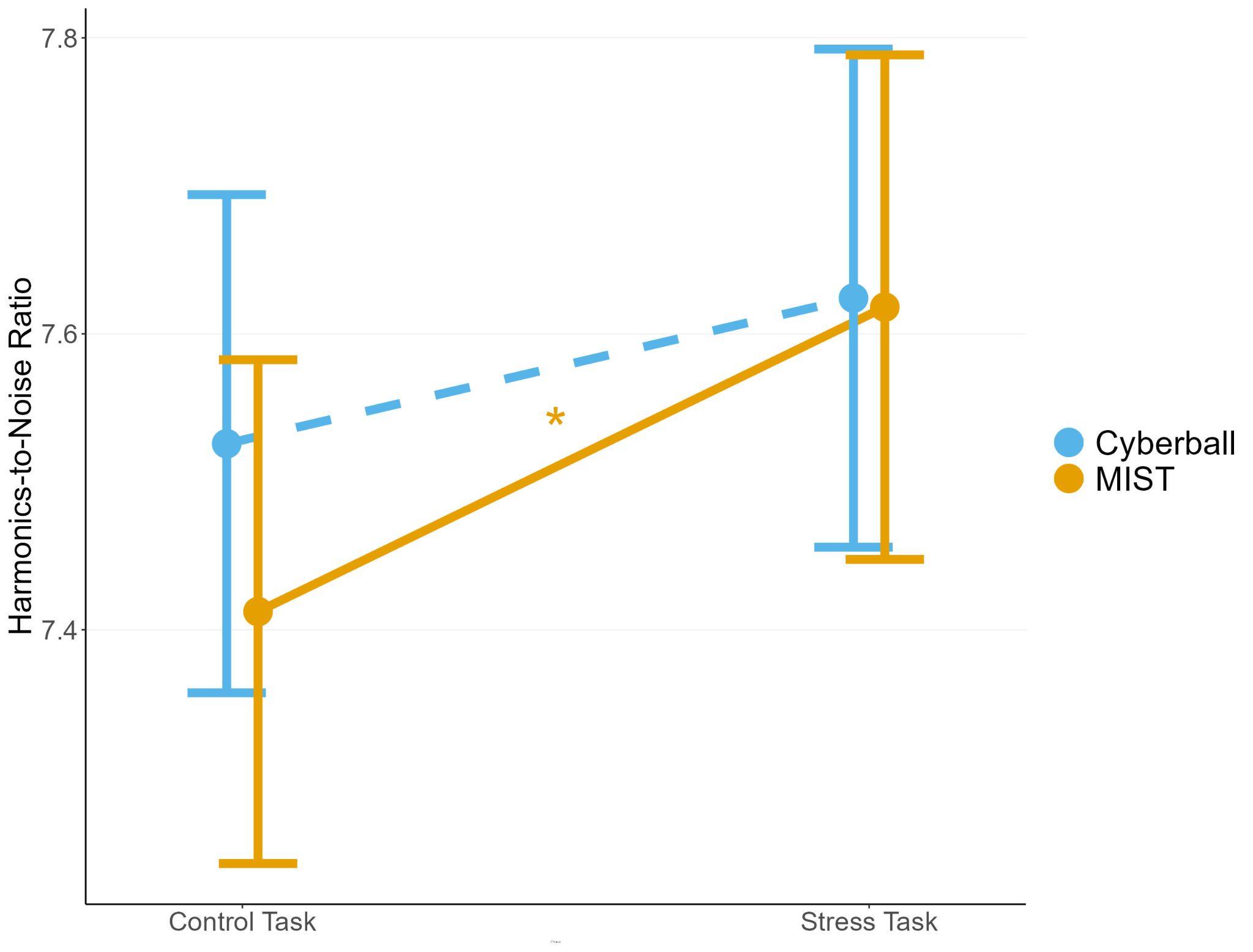


## 7.8) Shimmer

**Formula:** shimmerLocaldB_sma3nz_amean ~ fileNum * taskType + Sex + (1|participantNum)

**Anova:**

Analysis of Deviance Table (Type III Wald chisquare tests)

Response: shimmerLocaldB_sma3nz_amean

Chisq Df Pr(>Chisq)

(Intercept) 2617.9744 1 < 2.2e-16 ***

fileNum 1.5846 1 0.2081

taskType 25.8647 1 3.662e-07 ***

Sex 0.2809 1 0.5961

fileNum:taskType 1.3453 1 0.2461

---

Signif. codes: 0 ‘***’ 0.001 ‘**’ 0.01 ‘*’ 0.05 ‘.’ 0.1 ‘ ’ 1

**Figure:**


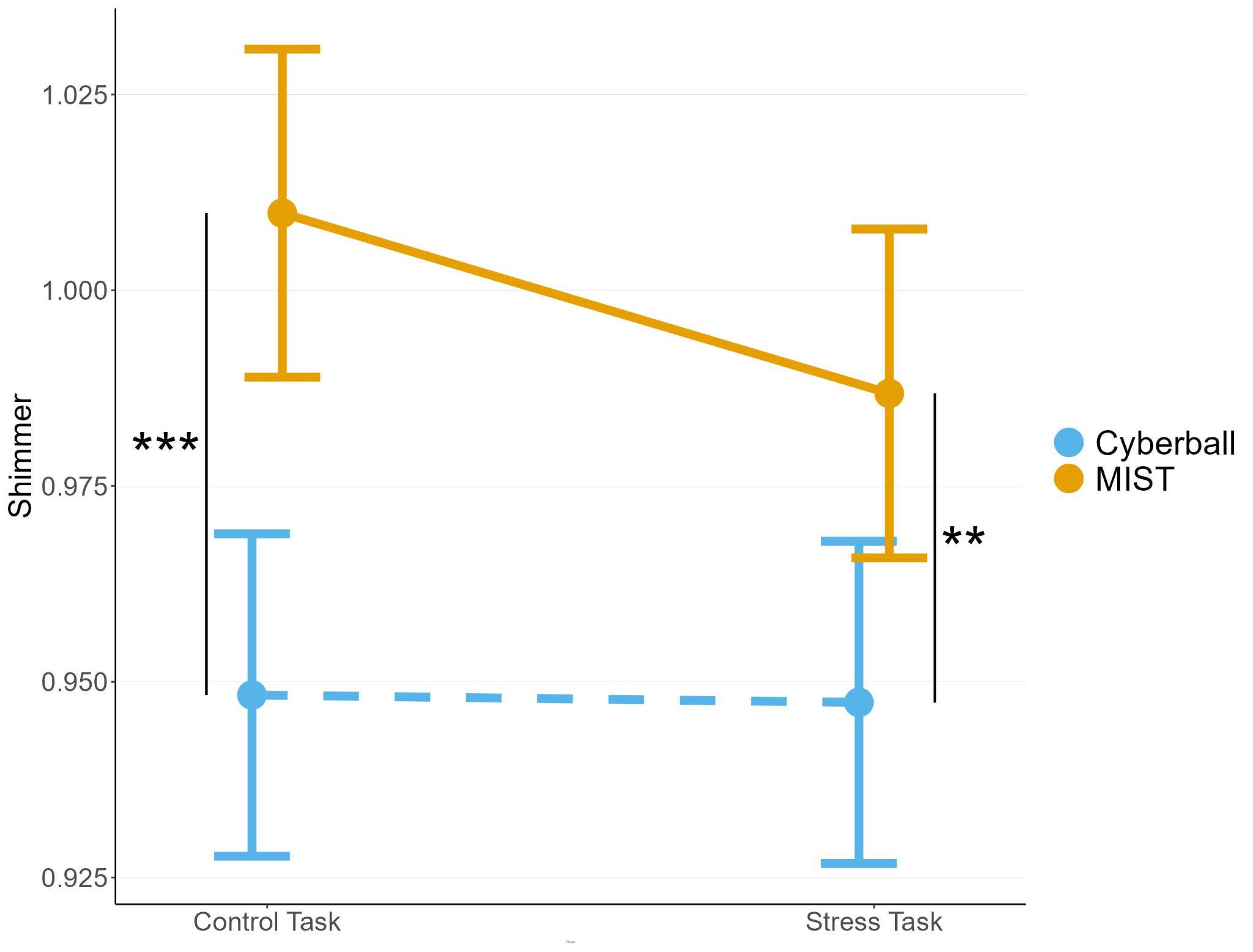


## 7.9) Jitter

**Formula:** jitterLocal_sma3nz_amean ~ fileNum * taskType + (1|participantNum)

**Anova:**

Analysis of Deviance Table (Type III Wald chisquare tests)

Response: jitterLocal_sma3nz_amean

Chisq Df Pr(>Chisq)

(Intercept) 1012.8052 1 < 2.2e-16 ***

fileNum 11.1673 1 0.0008325 ***

taskType 2.8528 1 0.0912171 .

fileNum:taskType 2.1522 1 0.1423676

---

Signif. codes: 0 ‘***’ 0.001 ‘**’ 0.01 ‘*’ 0.05 ‘.’ 0.1 ‘ ’ 1

**Figure:**


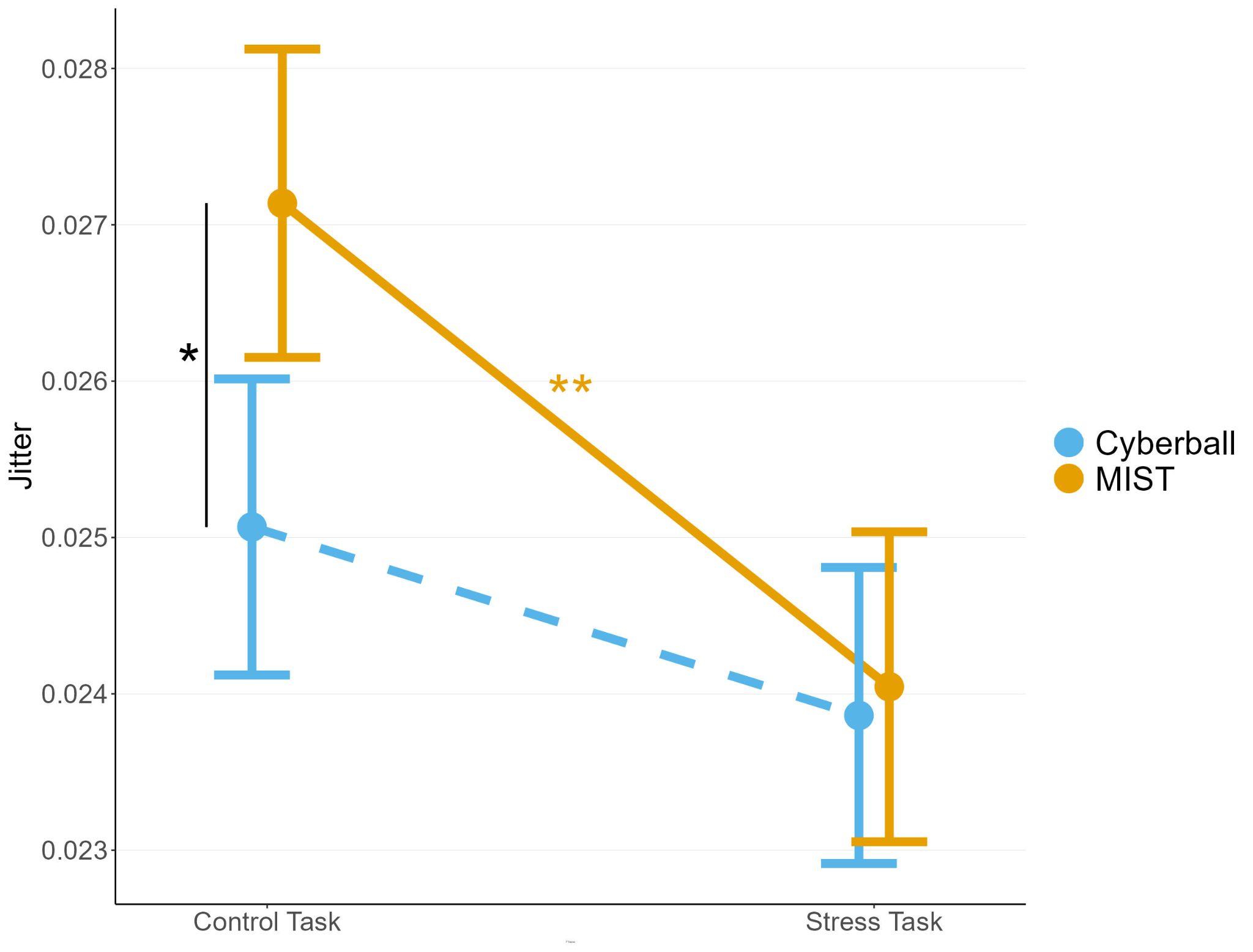


## 7.10) Correlation Table


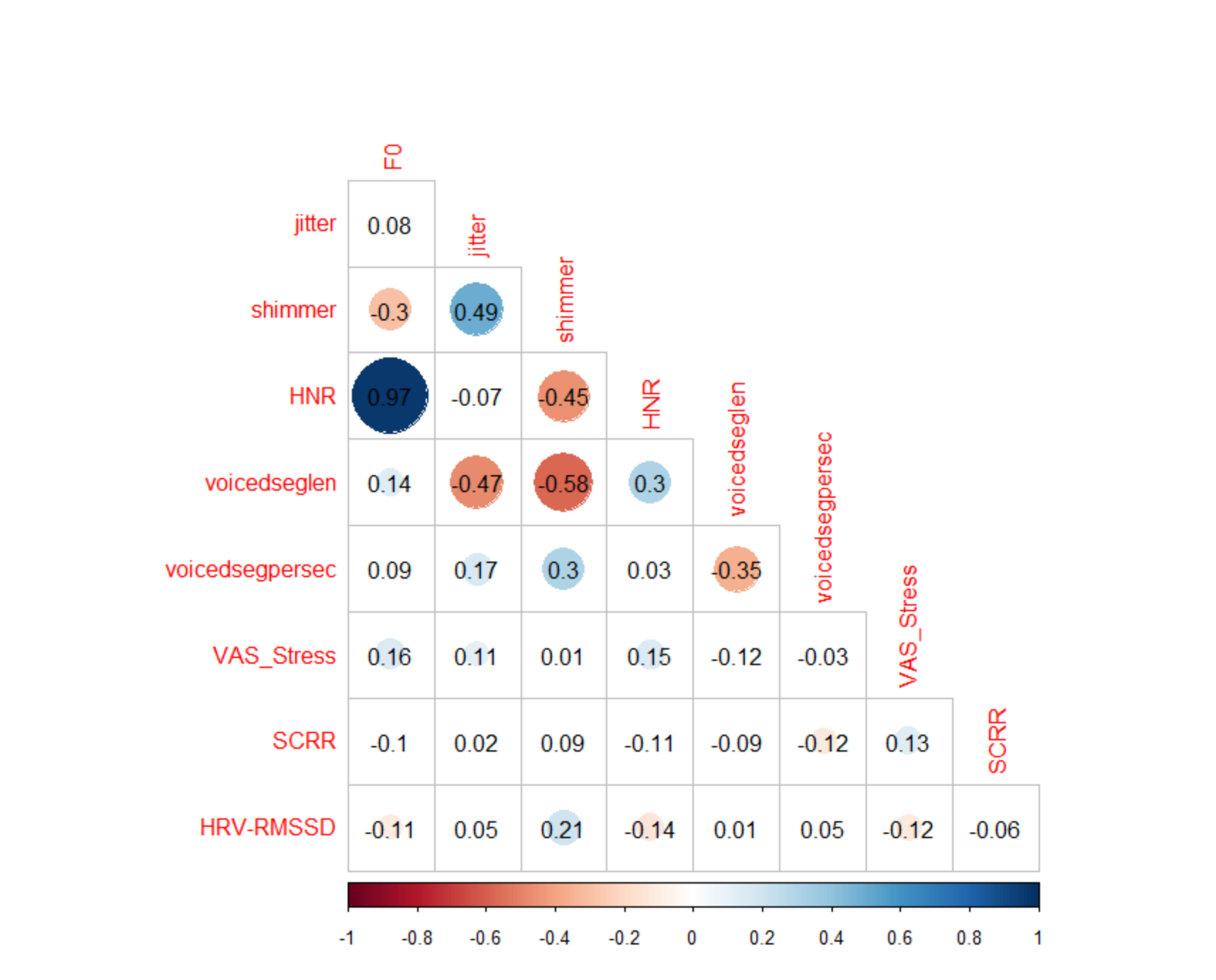


This table shows correlation coefficients between all included speech features, as well as self-reported stress (VAS_Stress) and psychophysiological measures; SCRR and HRV-RMSSD. Blank squares with just a correlation coefficient indicate non-significance, whereas colored circles indicate significance as well as direction (blue; positive, red; negative) and strength (darkness).
